# Chemically modified gelatin derivatives: synthetic strategies, physicochemical properties, biodegradation, drug delivery and biomedical applications

**DOI:** 10.3389/fphar.2026.1936037

**Published:** 2026-09-30

**Authors:** Amit Kunwar, J. Aishwarya, Koushik Majee

**Affiliations:** 1 Life Sciences Discipline, Homi Bhabha National Institute, Mumbai, India; 2 Radiation and Photochemistry Division, Bhabha Atomic Research Centre, Mumbai, India

**Keywords:** Gelatin, extraction, molecular structure, chemical modification, physicochemical properties, biomedical applications

## Abstract

Gelatin, the hydrolysate of collagen, has been used as a food ingredient as well as a pharmaceutical excipient since ancient times. Of late, it has attracted renewed interest as a constituent of drug delivery systems (DDS) because of its amicability to easily transform into various structural forms such as hydrogel, nanoparticles, and microsphere among others. Further, to meet the requirements for the fabrication of DDS, researchers have attempted various kinds of chemical modifications of the native gelatin. Some of the major chemical modification strategies of gelatin include amination, thiolation, PEGylation, acrylation and selenation, among others. Increasing evidences so far have established that chemically modified derivatives of gelatin exhibit superior physicochemical properties compared to native gelatin to support drug delivery applications such as higher drug loading efficiency and improved drug release kinetics, biocompatibility, biodegradability and pharmacokinetic profile. Accordingly, chemically modified derivatives of gelatin have been preferred over native gelatin for biomedical applications. Moreover, the growing popularity of chemically modified derivatives of gelatin has prompted their commercial production. In view of these considerations, a dedicated review article summarizing the biochemistry of gelatin, various synthetic strategies used for the preparation of chemically modified derivatives of gelatin, mechanistic discussion on the superiority of the Physicochemical properties of gelatin derivatives along with recent progress and concerns linked to their biomedical applications, is warranted. The present review article addresses this objective by providing a comprehensive, critical, and up-to-date summary of chemically modified derivatives of gelatin.

## 1 Introduction

Proteins are one of the most critical biomolecules, which are responsible for all biological functions/activities in living organisms. Proteins are formed within the cells of all living organisms through a complex biological process known as translation. Briefly, translation involves the joining of amino acids by a peptide bond to form a polypeptide chain. There are twenty natural amino acids, which, depending on the genetic sequence, can combine in varying permutations to generate various proteins (Hoerter and Ellis, 2025; Alberts et al., 2002). About structure, proteins can have four levels of organisation, namely, primary, secondary, tertiary, and quaternary structures (Hoerter and Ellis, 2025; Alberts et al., 2002). The primary structure of a protein represents the linear polypeptide chain stabilized through a peptide bond, which is formed between the carboxylic and amino groups of successive amino acids. The secondary structure of protein represents the folded structure stabilized through intrachain disulfide linkage and hydrogen bonds formed between the functional groups of side chains of amino acid residues. The tertiary structure generally represents the highest order of organisation of a protein with a supercoiled structure stabilized by hydrophobic and Vander wall interactions. Proteins to be functionally active need to acquire the tertiary structure. Further, the quaternary structure is seen in the case of proteins having more than one polypeptide chain. In such proteins, quaternary structure involves the interchain interaction between two or more polypeptide chains to form the functionally active protein. In a true sense, a polymer should contain the repeating units called monomer and thereby proteins containing amino acids as the repeating units, are classified as bioploymer. In recent years, proteins have attracted the attention of material chemists for the design of biomaterials for wide-ranging applications, including but not limited to tissue engineering, drug delivery systems (DDS) and biosensing (Singh et al., 2019; Sohail et al., 2026; Tunuhe et al., 2025; Abascal and Regan, 2018; Gregorio et al., 2025). The advantage of using proteins primarily relies on their ability to perform a specific function or recognition, or self-assembly, due to their anchoring structures and high mechanical strength, as discussed above. Additionally, proteins are also amicable for custom functionalization through chemical modification due to the availability of functional groups such as free thiol, carboxylic and amine groups in their backbone (Singh et al., 2019; Sohail et al., 2026; Tunuhe et al., 2025; Abascal and Regan, 2018; Gregorio et al., 2025). Moreover, peptides of desired amino acid sequence can be designed through genetic engineering as well as chemical synthetic approaches (Singh et al., 2019; Sohail et al., 2026; Tunuhe et al., 2025; Abascal and Regan, 2018; Gregorio et al., 2025). Nevertheless, proteins as biomaterials also have limitations. One such major limitation is the immunogenicity, which restricts their use for biomedical applications (Singh et al., 2019; Sohail et al., 2026; Tunuhe et al., 2025; Abascal and Regan, 2018; Gregorio et al., 2025). Accordingly, the most important criterion while designing the protein-based biomaterials is the choice of protein, which should be either non-immunogenic or weakly immunogenic (Agnieray et al., 2021). In this context, gelatin, the hydrolysate derived from collagen of animal body parts, has emerged as the most widely used protein for drug delivery and biomedical applications (Su and Wang, 2015; Milano et al., 2023; Jia et al., 2024; Zhang and Wang, 2024; Mohanto et al., 2023; Mikhailov, 2023). Historically, the usage of jelly prepared from fish and meat broths as a food ingredient dates back to as early as the 10th century BC (Mikhailov, 2023). However, it was during the 15^th^century that gelatin began to be commercially produced from the cattle hooves and waste of meat production factories by the boiling method. Later, French industrialist Denis Papin in 1681 improved the methodology of gelatin preparation by using boiling bone as the raw material (Mikhailov, 2023). Subsequently, another French researcher, Jean-Pierre-Joseph d’Arcet (1812), introduced the use of hydrochloric acid or acid digestion along with a boiling step for the extraction of gelatin from the animal body parts (Mikhailov, 2023). The use of acid digestion, along with boiling, substantially improved the yield and purity of gelatin. Slowly, gelatin gained popularity as a cheap source of protein for consumption. While gelatin continues to be the most widely used gelling agent in the food industry, it also received attention for other applications, particularly in the field of pharmaceuticals as a carrier of drug molecules. A wide range of gelatin-based carriers, such as capsules, macroparticles, nanoparticles, emulsions, gels, etc., are very well documented in the literature for drug delivery applications. Subsequently, a lot of work has also been done on the covalent modification of gelatin with desired chemical functionality to tune its gelling, mechanical, and drug delivery properties. Over the past few years, dedicated review articles have been published on the biomedical applications of gelatin *per se*, its various carrier forms, and its derivatives (Su and Wang, 2015; Milano et al., 2023; Jia et al., 2024; Zhang and Wang, 2024; Mohanto et al., 2023; Mikhailov, 2023; Elzoghby, 2013). However, to the best of our knowledge, the previous reviews have not comprehensively addressed the diverse synthetic/biochemical approaches involved in the preparation of gelatin derivatives, nor have they discussed several newly developed derivatives, such as azido- and selenium-functionalized gelatin. These limitations highlight the need for an updated review that can serve as a comprehensive reference for researchers engaged in the research and development of gelatin derivatives and facilitate a better understanding of this rapidly evolving field. Accordingly, the present review aims to address these knowledge gaps. It initially discusses the isolation and extraction strategies, as well as the biochemistry of native gelatin. This is followed by dedicated sections on the major chemical derivatives of gelatin, including aminated gelatin, methacrylated gelatin, PEGylated gelatin, thiolated gelatin, azido gelatin, and seleno gelatin. Each section provides an up-to-date discussion of the respective synthesis methods, the effects of the degree of substitution on physicochemical properties, functionality, and biodegradability, and the biochemical basis underlying the superiority of these derivatives over native gelatin for drug delivery applications. In addition, a dedicated future perspective section highlights the limitations associated with chemically modified gelatins and discusses potential strategies to overcome these challenges.

## 2 Gelatin

Gelatin is the hydrolysed product of collagen, one of the most abundant (∼30% of total protein) structural proteins in animals (Eastoe and Ramachandran, 1976). The collagen extracted from different body parts (such as skin, bone, cartilage, tendon, ligaments, scales, etc.) of a wide range of animal species (like bovine, porcine, equine and fish, among others) is used for the preparation of gelatin by following the methodology as shown in Figure 1 (Mikhailov, 2023; Elzoghby, 2013; Eastoe and Ramachandran, 1976; Alleavitch et al., 1989; Mahmood et al., 2016; Schrieber and Gareis, 2007; Cole and Francis, 2000; Nur Hanani, 2016). The initial step of the preparation of gelatin involves extracting collagen from the animal tissue by removing fat and the major contaminant or “ballast” proteins, such as albumin, globulin, mucoids, etc. To remove the contaminant proteins, the animal tissue, depending on its complexity, is treated with either hydrochloric acid (HCl) or slaked lime (Ca(OH)_2_), followed by neutralization. The treatment with HCl or Ca(OH)_2_ leads to specific degradation of albumin/globulin with a minor change in the structural integrity of collagen. Subsequently, animal tissue is subjected to the boiling water treatment to get rid of fat. The resulting tissue extract is rich in collagen protein and is further processed for the preparation of gelatin. Briefly, the collagen extract is subjected to hydrolysis by using acid or base to produce gelatin. The gelatin produced through acid hydrolysis is known as type A, whereas that produced through alkaline hydrolysis is classified as type B (Mikhailov, 2023; Elzoghby, 2013; Eastoe and Ramachandran, 1976; Alleavitch et al., 1989; Mahmood et al., 2016; Schrieber and Gareis, 2007; Cole and Francis, 2000; Nur Hanani, 2016). Of late, gelatin is also being produced through enzymatic hydrolysis of collagen (Mikhailov, 2023; Elzoghby, 2013; Eastoe and Ramachandran, 1976; Alleavitch et al., 1989; Mahmood et al., 2016; Schrieber and Gareis, 2007; Cole and Francis, 2000; Nur Hanani, 2016). The acid hydrolysis is preferred for the processing of simpler (or low average molecular weight) collagen, such as that derived from porcine skin. The commonly employed acids in this process are HCl, sulfuric acid (H_2_SO_4_) or phosphoric acid (H_3_PO_4_) (Mikhailov, 2023; Elzoghby, 2013; Eastoe and Ramachandran, 1976; Alleavitch et al., 1989; Mahmood et al., 2016; Schrieber and Gareis, 2007; Cole and Francis, 2000; Nur Hanani, 2016). The alkaline hydrolysis is preferred for the processing of complex (or high average molecular weight) collagen, such as that derived from bovine hides. It usually takes a longer duration than acid hydrolysis and involves incubating collagen with Ca(OH)_2_ (Mikhailov, 2023; Elzoghby, 2013; Eastoe and Ramachandran, 1976; Alleavitch et al., 1989; Mahmood et al., 2016; Schrieber and Gareis, 2007; Cole and Francis, 2000; Nur Hanani, 2016). In comparison to acid and alkaline hydrolysis, the enzymatic hydrolysis is a superior processing method as it ensures minimal degradation and higher purity of gelatin (Mikhailov, 2023; Elzoghby, 2013; Eastoe and Ramachandran, 1976; Alleavitch et al., 1989; Mahmood et al., 2016; Schrieber and Gareis, 2007; Cole and Francis, 2000; Nur Hanani, 2016). Additionally, it also takes a shorter processing time as compared to acid/alkaline hydrolysis. Following hydrolysis, the collagen extract is subjected to filtration, which gives the concentrated gelatin solution. Subsequently, the gelatin concentrate is passed through sterilization process (heating at 130^∘^ C under pressurized environment) in order to make it free from endotoxins and other life forms, mainly the microbes and viruses. Further, the gelatin concentrate is solidified by evaporation, dried and crushed into powder form (Mikhailov, 2023; Elzoghby, 2013; Eastoe and Ramachandran, 1976; Alleavitch et al., 1989; Mahmood et al., 2016; Schrieber and Gareis, 2007; Cole and Francis, 2000; Nur Hanani, 2016). Finally, the gelatin powder is passed through molecular sieves in order to achieve homogeneity as well as to segregate it according to the bloom strength or average molecular weight. The bloom strength is also known as gelling strength and is proportional to the average molecular weight of gelatin. The bloom strength of any polymeric material is expressed in terms of a bloom unit, which is equivalent to the force needed to cause depression of the gel formed by a known concentration of polymer by a distance of 4 mm (Borker et al., 1966; Osorio et al., 2007). The bloom number of a polymeric material is determined using a gelometer. According to bloom strength, the gelatin has been classified mainly into three types, such as low, medium and high bloom gelatin (Mikhailov, 2023; Elzoghby, 2013; Eastoe and Ramachandran, 1976; Alleavitch et al., 1989; Mahmood et al., 2016; Schrieber and Gareis, 2007; Cole and Francis, 2000; Nur Hanani, 2016; Borker et al., 1966; Osorio et al., 2007; Duthen et al., 2018). The low bloom gelatin is represented by bloom units or numbers ranging from 50 to 125 and is proportional to average molecular weight in the range of 20–25 KDa (Figure 1). The medium bloom gelatin is represented by bloom units or numbers ranging from 175 to 225 and is proportional to average molecular weight in the range of 40–50 KDa (Figure 1). Whereas, the high bloom gelatin is represented by bloom units or numbers ranging from 225 to 325 and is proportional to average molecular weight in the range of 50–100 KDa (Figure 1).

**FIGURE 1:**
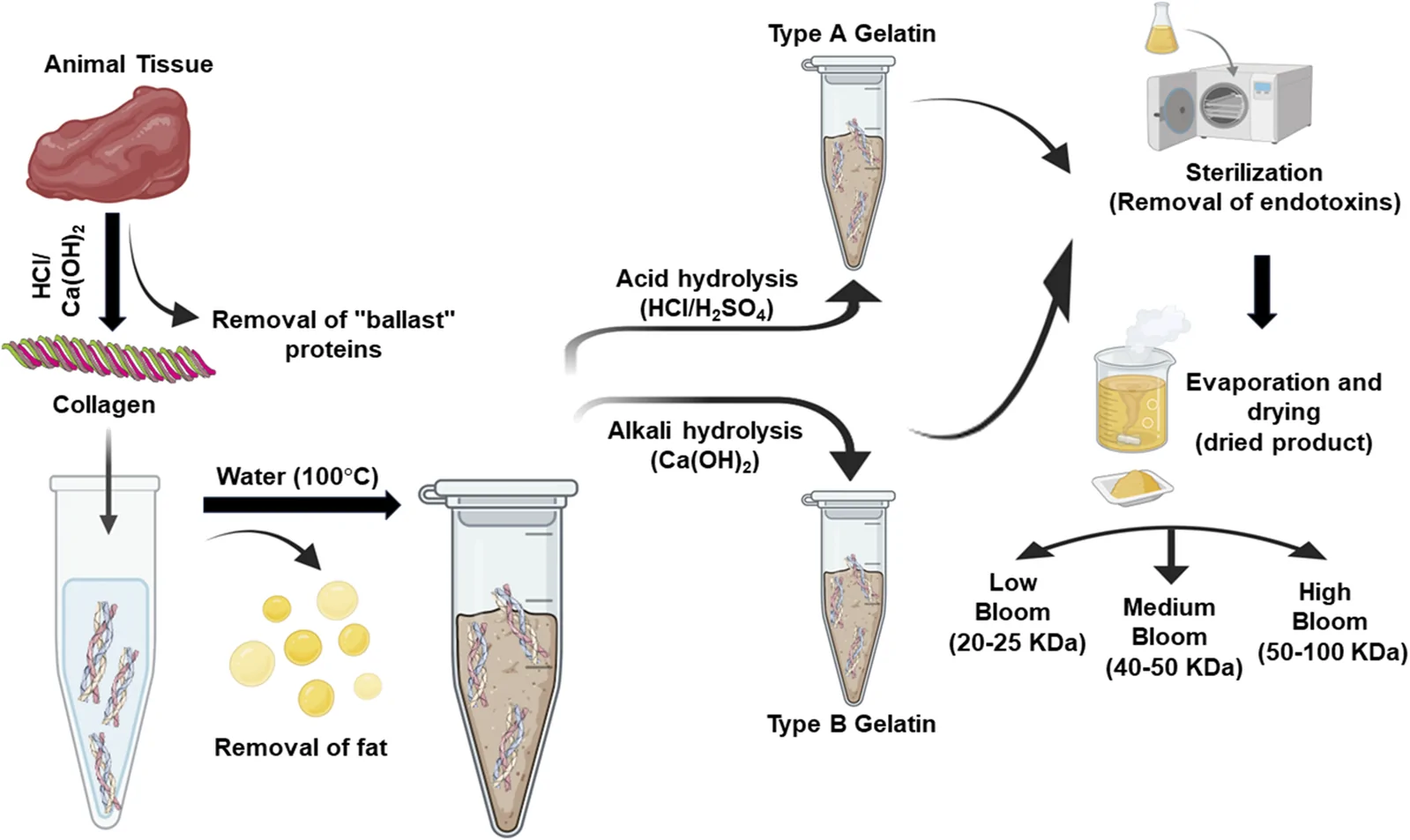
Schematic showing the extraction procedure and classification of gelatin from animal tissues. Illustrations made using Biorender.com.

Further, the amino acid composition of gelatin comprises 18 out of the 20 natural amino acids except cysteine and tryptophan (Mikhailov, 2023; Liu et al., 2015; Neuman, 1949; Eastoe, 1955). About two-thirds of the total amino acids present in gelatin are hydrophobic residues. Despite this, gelatin is highly soluble in water and is practically insoluble in organic solvents. The amino acid composition and the major structural forms of gelatin are shown in Figure 2. The most abundant amino acid in gelatin is glycine (∼22%), followed by proline (∼12%) and hydroxyproline (∼12%). Being the hydrolysate of collagen, the molecular structure of native gelatin is similar to that of collagen (Ramachandran et al., 1955; Rich and Crick, 1955). Briefly, a gelatin molecule exhibits a triple helical structure comprising three polypeptide chains (Mikhailov, 2023; Cowan and McGavin, 2020; Veis et al., 1961; Boedker and Doty, 1954; Groome and Clegg, 1965; Chen et al., 1991; Hulmes et al., 1973; Darvish, 2022). Of the three polypeptide chains, the two share an exactly similar amino acid sequence as well as the molecular weight and are denoted as alpha 1 (α1) chain (Mikhailov, 2023; Cowan and McGavin, 2020; Veis et al., 1961; Boedker and Doty, 1954; Groome and Clegg, 1965; Chen et al., 1991; Hulmes et al., 1973; Darvish, 2022). The third polypeptide is denoted as alpha 2 (α2) chain and its amino acid sequence is not yet fully understood (Mikhailov, 2023; Cowan and McGavin, 2020; Veis et al., 1961; Boedker and Doty, 1954; Groome and Clegg, 1965; Chen et al., 1991; Hulmes et al., 1973; Darvish, 2022). A gelatin molecule typically contains the three polypeptide chains in the stoichiometry of α1_2_α2 (Mikhailov, 2023; Cowan and McGavin, 2020; Veis et al., 1961; Boedker and Doty, 1954; Groome and Clegg, 1965; Chen et al., 1991; Hulmes et al., 1973; Darvish, 2022). However, gelatin from some sources can also have the stoichiometry of α1_3_ (Mikhailov, 2023; Cowan and McGavin, 2020; Veis et al., 1961; Boedker and Doty, 1954; Groome and Clegg, 1965; Chen et al., 1991; Hulmes et al., 1973; Darvish, 2022). The three polypeptide chains are wrapped around each other to form the right-handed helical structure, which is stabilized by interchain hydrogen bonding. Notably, gelatin also exhibits a unique feature of glycine residue repeating at every third position after the 17th amino acid in its α1 polypeptide chain (Mikhailov, 2023; Cowan and McGavin, 2020; Veis et al., 1961; Boedker and Doty, 1954; Groome and Clegg, 1965; Chen et al., 1991; Hulmes et al., 1973; Darvish, 2022). Accordingly, the structure of gelatin is represented as the repeats of a tripeptide sequence (Gly-A-B). Here, the A and B positions are commonly occupied by proline and hydroxyproline residues, respectively (Mikhailov, 2023; Cowan and McGavin, 2020; Veis et al., 1961; Boedker and Doty, 1954; Groome and Clegg, 1965; Chen et al., 1991; Hulmes et al., 1973; Darvish, 2022). Additionally, gelatin also contains the unevenly distributed arginine–glycine–aspartic acid (RGD) tripeptide sequence, which is responsible for its mucoadhesive property through recognition by integrin receptors (Mikhailov, 2023). Gelatin, during its preparation from collagen, loses the native structure due to separation of constituent polypeptide chains and is reorganised as a single α chain or covalently linked two α chains (known as β chain) or covalently linked three α chains (known as γ chain) (Mikhailov, 2023; Haug et al., 2004; Olsen, 1932; Guo et al., 2003; Stainsby et al., 1977). Further, depending on the concentration and temperature, the polypeptides chains of gelatin undergo intra and interchain hydrogen bonding to form various kinds of secondary structures such as non-looped single-strand helix, non-looped double-strand helix, non-looped triple-strand helix, single looped double-strand helix, single looped triple-strand helix and double lopped triple-strand helix as the nucleation centre ultimately leading to a dense network of coils known as gel (Mikhailov, 2023; Haug et al., 2004; Olsen, 1932; Guo et al., 2003; Stainsby et al., 1977; Borchard and Burg, 2007). Among the above secondary structures, non-looped single-strand helix, single looped double-strand helix and double looped triple-strand helix formed by a single α chain represent the first order of structural organisation (Figure 2). The non-looped double-strand helix and single looped triple-strand helix formed by the interaction of two α chains represent the second order of structural organisation (Figure 2). Whereas, a non-looped triple-strand helix formed by the interaction of three α chains is recognised as the third order of structural organisation (Figure 2). The dilute solution of gelatin predominantly exhibits the lower order of organisation. However, above the critical concentration (usually ∼1% w/v), the aqueous solution of gelatin shows thermo-responsive gelation behaviour characterised by solid gel formation at temperatures below ∼30 °C or sol transition at temperatures >40 °C (Borchard and Burg, 2007).

**FIGURE 2:**
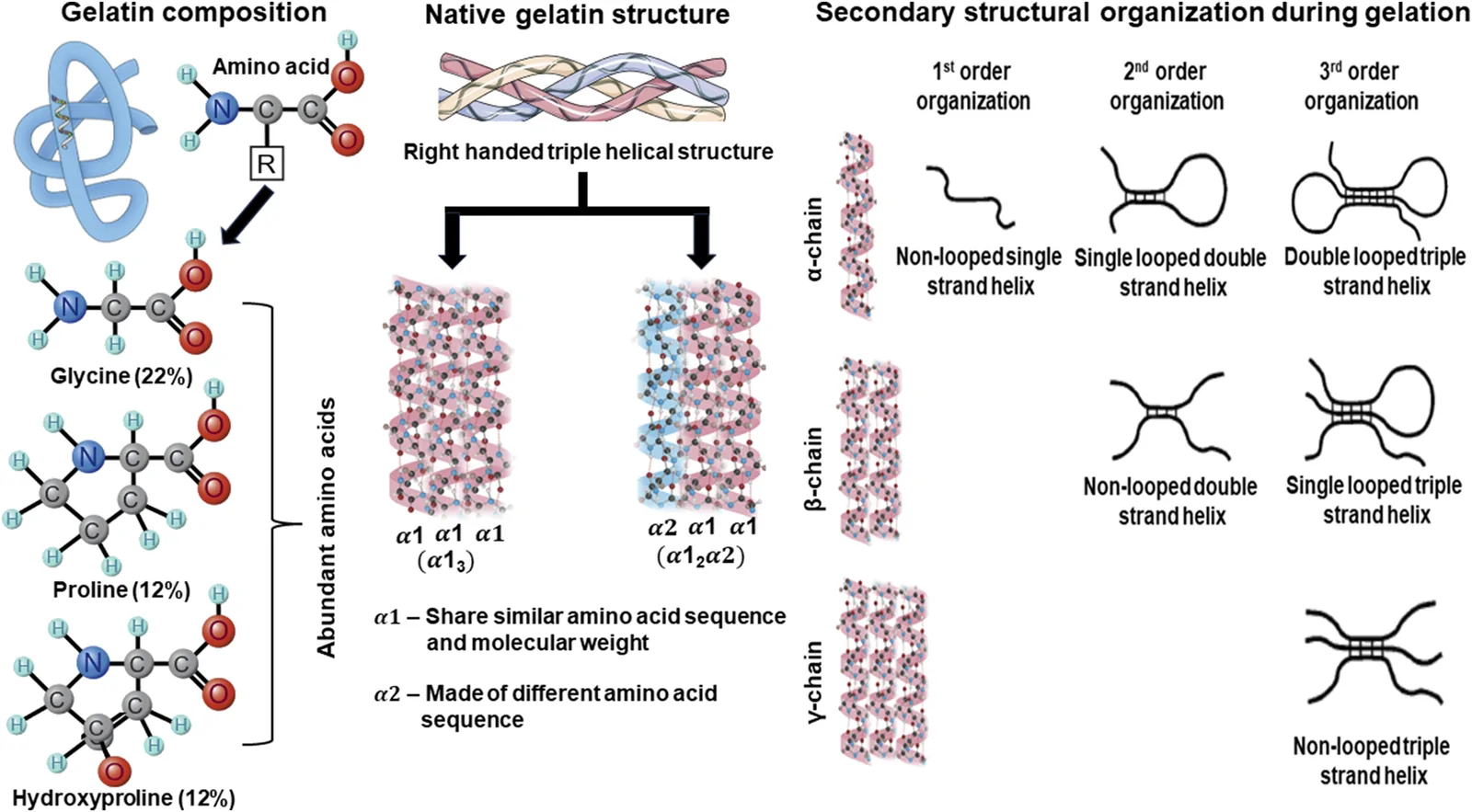
Schematic showing the various levels of structural organisation of gelatin. Illustrations made using Biorender.com.

The physicochemical properties of gelatin usually vary depending on the source of its origin (Table 1). Gelatin, like any other protein, is characterised by its isoelectric point (IEP). The IEP of gelatin is determined by the number of free carboxylic (-COOH), free amine (-NH_2_), amide (-CONH_2_) and guanidino ((NH_2_)_2_C = NH) groups present in the side chain of its amino acid residues. Depending on the processing method, the content of amino acid residues containing the above function groups varies significantly and thereby alters the IEP of gelatin. For instance, acid hydrolysis produces gelatin with a higher content of amide group-containing residues such as asparagine and glutamine, and thereby shifting the IEP of type A gelatin towards higher pH values (7.0–9.5) (Mikhailov, 2023; Cole and Francis, 2000; Liu et al., 2015; Neuman, 1949; Eastoe, 1955). On the other hand, alkaline hydrolysis leads to conversion of the amide group of asparagine and glutamine into carboxylic group, thereby increasing the content of carboxylic group in gelatin. Accordingly, the IEP of type B gelatin is shifted towards lower pH values (4.7–5.5) (Mikhailov, 2023; Cole and Francis, 2000; Liu et al., 2015; Neuman, 1949; Eastoe, 1955). Another important parameter, such as the viscosity of gelatin, also varies as a function of concentration and temperature (Schrieber and Gareis, 2007; Osorio et al., 2007; Haug et al., 2004; Stainsby et al., 1977). For instance, the viscosity of gelatin decreases with an increase in temperature at a particular concentration due to the denaturation of its polypeptide chains. Alternatively, the viscosity of gelatin at a particular temperature increases with increasing concentration due to the higher order of the structural arrangement of its polypeptide chains. The typical gel strength and the gelling point of type A and type B gelatins are listed in Table 1. Further, mammalian-derived gelatin exhibits a melting point in the range of 28 °C–31 °C, whereas those from fish origin in the range of 11 °C–28 °C (Schrieber and Gareis, 2007; Osorio et al., 2007; Haug et al., 2004; Stainsby et al., 1977).

**TABLE 1:** Physicochemical properties of gelatin obtained from various sources.

Type of gelatin	Source	IEP	Gel stregth	Gelling point	Melting point
Type A	Low average molecular weight collagen (Porcine, Equine and Fish)	pH ∼7.0–9.5	70–270 Bloom	8 °C–25 °C	11 °C–28 °C
Type B	High average molecular weight collagen (Bovine)	pH ∼4.7–5.5	100–300 Bloom	20 °C–25 °C	28 °C–31 °C

## 3 Chemically modified gelatin

The increasing utility of gelatin as a carrier of pharmaceutical formulations has paved the way for the emergence of various chemically modified derivatives of gelatin. The main rationale for the chemical modification of gelatin has been to make it suitable for further functionalization with desired utility groups or to modulate its physicochemical properties such as viscosity, gel strength, adhesiveness, melting point and mechanical strength, among others, to improve its utility. The synthetic approaches used for preparing some of the common gelatin derivatives (Figure 3) and their biomedical applications are discussed in the following sections.

**FIGURE 3:**
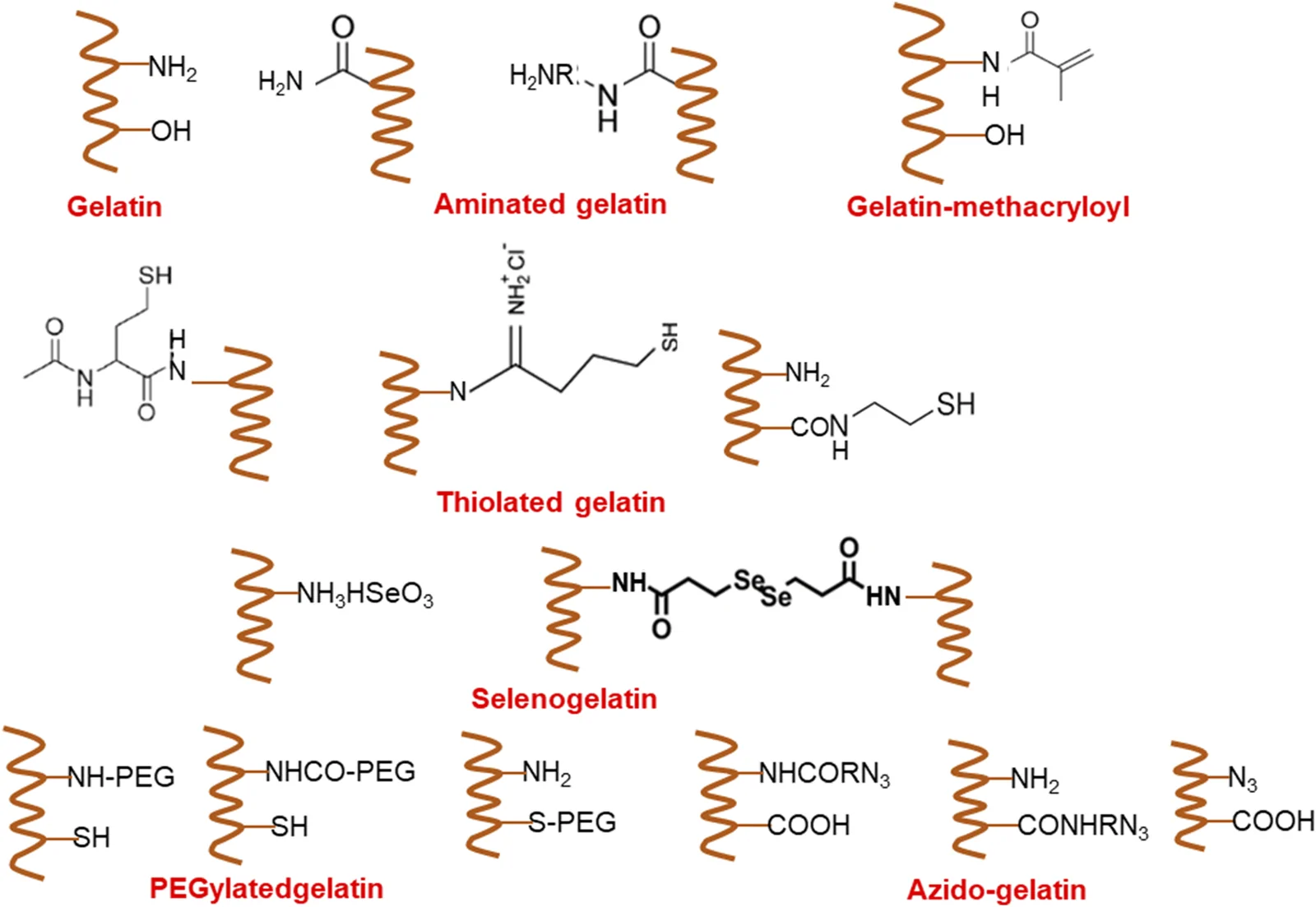
Commonly developed synthetic derivatives of gelatin.

### 3.1 Aminated gelatin

The native gelatin usually contains more of the carboxylic group than the amine group. For instance, type A gelatin contains about ∼78–80 mmol of free carboxylic group per 100 g of protein. Whereas, type B gelatin contains about ∼100–115 mmol of free carboxylic group per 100 g of protein. On the contrary, gelatin on average contains ∼30 mmol of free amine content per 100 g of protein (Duggan et al., 2015). The amination is performed to increase the amine content in gelatin so that it can be preferably used for conjugation with carboxyl group-containing utility groups. Type B gelatin is specifically preferred for amination reaction due to its higher carboxylic acid content. The basic strategy of synthesizing aminated gelatin involves either converting the free carboxylic (-COOH) groups into the corresponding carboxamide (-CONH_2_) groups or adding amine groups (-NH_2_) to the free carboxylic groups of the aspartic and glutamic acid residues of gelatin without altering other residues. Additionally, the synthetic process should not cause any interchain crosslinking of the gelatin. The method of the chemical modification of the carboxylic group in the protein was first reported by Hoare and Koshland in 1966 (Hoare and Koshland, 1966). Briefly, this process involved activating the free carboxylic group of the protein with water-soluble carbodiimide at pH ∼5, followed by reaction with nucleophilic modifying reagent to yield the desired product. This type of reaction in chemistry is well documented as carbodiimide coupling. Later on, several researchers have employed this methodology with slight modifications to synthesize the aminated gelatin. For instance, Hayashi et al. performed the amination of gelatin B by using 1-ethyl-3-(3-dimethylaminopropyl) carbodiimide (EDC) as the activating carbodiimide and ammonium sulphate as the nucleophilic modifying reagent (Hayashi et al., 1990). The reaction was carried out by incubating gelatin with saturated ammonium sulphate solution in the presence of EDC at 40^∘^ C for 40 min. During the reaction, the pH of the reaction mixture was maintained at 4.7 by using 2N HCl. The EDC was varied from 0.12 to 0.72 M equivalents of carboxyl group of gelatin to achieve an increasing degree of amination. The reaction was stopped by dropwise addition of acetic acid, and the product (aminated gelatin) was purified through dialysis. The degree of amination of gelatin was determined by measuring the release of ammonia following treatment with 6N HCl. The detailed physicochemical characterisation of the reaction product confirmed the successful conversion of the free carboxylic groups of gelatin into carboxyamide groups without any interchain crosslinking as well as modification of residues other than aspartic and glutamic acids. The degree of amination increased with increasing molar ratio of EDC versus carboxylic groups in the gelatin. The 0.72 M ratio of EDC versus carboxylic groups could achieve amination of ∼46 residues per 1000 amino acid of gelatin, which corresponded to about ∼40 percent of total carboxylic groups (aspartic acid and glutamic acid) in gelatin B. As expected, aminated gelatin B exhibited a higher IEP than non-amidated gelatin B. Further, gelatin B with an increasing degree of amination showed a decrease in the gel strength. Similarly, the viscosity of the aminated gelatin B was also lower than that of the non-aminated gelatin B. Within the aminated group, the viscosity increased with increasing degree of amination. Moreover, this study also established that the ability of gelatin B to form a triple helical structure during gelation is hindered by amination, resulting in poor gel strength. Therefore, the study concluded that aminated gelatin *per se* may not be an effective precursor for forming a hydrogel. However, with further suitable functionalization, even the aminated gelatin can form gels for desired biomedical applications. In another study, Wang et al. performed the amination of gelatin A (IEP:9) by reacting it with an excess of 1,2-ethylenediamine (EDA) in the presence of EDC in the phosphate buffer of pH ∼5 (Wang et al., 2000; Wang et al., 2001). The reaction was carried out at 37 °C for 1 h, followed by dialysis and freeze-drying to yield the aminated gelatin as the powder. The modified gelatin showed almost ∼2-fold higher free amine content (∼1.6 μ mol/mg protein) than the native non-aminated gelatin (∼0.8 μ mol/mg protein). Another approach, which has recently been reported for preparing the aminated gelatin, involved reaction of an aqueous solution of gelatin A with ∼2.5 weight equivalent of adipic acid dihydrazide (ADH) in the presence of EDC and 1-hydroxybenzotriazole hydrate (HOBt) for 24 h at a temperature and pH of 25^∘^ C and 5 respectively, followed by purification using dialysis (Lai and Hsieh, 2012; Morimoto et al., 2008). The ninhydrin assay was performed to quantify the free amine content of the modified gelatin. The physicochemical characterisation, coupled with the ninhydrin assay, validated the successful amination with the modified gelatin showing 48 free amine groups per molecule as compared to ∼33 free amine groups per molecule of non-aminated gelatin A. Moreover, the modified gelatin A showed a marginally increased molecular weight of 102 kDa as compared to 99 kDa of non-aminated gelatin A, confirming that binding of ADH to the carboxylic group of the aspartic and glutamic acid residues did not involve the interchain cross-linking. Subsequently, authors varied the weight ratio of gelatin A versus ADH as 1:3, 1:6 and 1:12 and determined the degree of amination. As expected, the results showed an increase in the degree of amination with increasing weight ratio of ADH versus gelatin. For instance, modified gelatin prepared using 1:3, 1:6, and 1:12 weight ratios of gelatin versus ADH exhibited the presence of about ∼50, ∼66, and ∼100 free amine groups per molecule, respectively, which is significantly higher than that of non-aminated gelatin A (∼33 per molecule). Together, these results established ADH as an effective agent for achieving the amination of gelatin and also the dependency of the degree of amination on the weight ratio of gelatin versus ADH. Furthermore, Duggan et al. has recently reported that the reaction temperature significantly influences the amination of gelatin through carbodiimide coupling (Duggan et al., 2015). Briefly, the authors performed the carbodiimide coupling of gelatin using EDC/EDA at room temperature (25^∘^ C) and at 37^∘^ C. The degree of amination was determined by quantifying the free amine content using the 2,4,6-trinitrobenzene sulphonic acid (TNBS) assay. The results indicated that the carbodiimide coupling reaction at 37^∘^ C yielded a lesser degree of amination (603.9 ± 53.0 μmol of amine per gram of gelatin) as compared to the similar reaction performed at room temperature (1210.4 ± 49.2 μmol of amine per gram of gelatin). Authors attributed the temperature-dependent degradation of gelatin as the reason for the lower degree of amination at 37^∘^ C. Notably, authors also observed that gelatin reaction with EDA in the absence of EDC also yielded higher amine content (452.5 ± 39.5 μmol of amine per gram of gelatin) than the native gelatin (267.7 ± 12.5 μmol of amine per gram of gelatin). The authors attributed this observation to the trapping of unbound EDA within the gelatin matrix during the reaction. Accordingly, the authors suggested incorporating reaction control groups (gelatin reaction with EDA without EDC) for the accurate determination of the degree of amination.

### 3.2 Thiolated gelatin

Thiolation of biopolymers improves their mucoadhesive property as it facilitates the formation of disulphide linkages between the polymeric material and mucosal layer (Gyarmati et al., 2013). Additionally, thiolation can also facilitate the formation of intrachain disulfide linkages and thereby can improve the mechanical strength as well as stimuli-responsive release characteristics of the polymeric material (Gyarmati et al., 2013). Accordingly, thiolated gelatin has been perceived as the superior material for various biomedical applications. The synthetic approach for chemically introducing the free sulfhydryl groups into the protein was first reported by Benesch and Benesch (1956 & 1958) (Benesch and Benesch, 1956; Benesch and Benesch, 1958). Interestingly, authors used gelatin as the model protein for thiolation in these studies. Initially, authors performed preliminary experiments using amino acid (like glycine) and established that the reaction of amino acid with N-substituted homocysteine thiolactone under mild conditions as possible, i.e., in aqueous solution, at low temperature and at moderate alkaline pH (∼9) introduced free sulfhydryl group at the amine group of amino acid through ring opening mechanism followed by formation of peptide bond (Benesch and Benesch, 1956). However, authors recognised the water insolubility of thiolactone at low temperature, the formation of homocysteinylhomocysteine bond and the hydrolytic splitting of thiolactone ring, resulting into formation of undesired products as the three major limitations of such reactions. To overcome these issues, authors evaluated a series of N-substituted homocysteine thiolactones as thiolating agent and established that N-acetyl homocysteine thiolactone (AHTL) was the most suitable reagent to achieve the higher degree of amino acid thiolation as it was water soluble, did not facilitate the formation of homocysteinylhomocysteine bond due to N-acetylation of its amine group and was also stable at alkaline pH required for facilitating the reaction. Although this methodology appeared ideal for the thiolation of amino acids, the application of this reaction for achieving protein thiolation was limited due to the requirement of alkaline conditions (ensuring that all the free amine groups of the amino acid residues are in the deprotonated (-NH_2_) rather than protonated (-NH_3_^+^) form) for obtaining good yields. Accordingly, in order to prevent the protein from undergoing denaturation or damage during the thiolation reaction, the authors further improved the methodology by introducing a slight modification (Benesch and Benesch, 1958). For instance, authors performed the reaction of gelatin (model protein) with AHTL in the presence of silver ions (Ag^+^). The rationale for using silver ions was to facilitate the reaction at near neutral pH of ∼7.5. The study indeed established that in the presence of silver ion the thiolation proceeded rapidly at room temperature in aqueous solution at pH 7.5 without damaging the native structure of gelatin. The reaction mechanism was explained as the formation of an insoluble complex of silver ions with AHTL, followed by nucleophilic attack by the complex on the free amine groups of the amino acid residues of gelatin, resulting in the thiolation. Moreover, silver ions also protected the sulfhydryl groups from oxidation after opening of the lactone ring, thereby improving the yield. The number of sulfhydryl groups introduced in the gelatin by this method varied over a wide range (∼10–∼30 groups) simply by changing the molar ratio (1–10 mol per amino group) of AgAHTL. Furthermore, authors suggested that it was also possible to introduce free sulfhydryl groups preferably at the α-amine group rather than the ε-amine group of amino acid residues by simply controlling the pH of the reaction mixture. Recently, Vlierberghe et al. reported the chemical modification of gelatin B (bloom strength:257) with a varying degree of thiolation employing 1- to 5-fold weight equivalents of AHTL in aqueous medium (Van Vlierberghe et al., 2011; Van Hoorick et al., 2021). Importantly, authors performed the reaction under alkaline conditions (carbonate buffer, pH ∼10) at 40^∘^ C for 3 hours without the addition of silver ions in the reaction mixture. Instead, authors added ethylene diamine tetraacetic acid (EDTA) in the reaction mixture to chelate any divalent metal ions, which could catalyse the oxidation of the free sulfhydryl group of the thiolated gelatin into disulfide linkages. The modified gelatin was purified through dialysis, and the extent of thiolation was quantified either directly by measuring the amount of free sulfhydryl groups using Ellman’s reagent (5,5′-dithio-bis-(2-nitrobenzoicacid) or DTNB) or indirectly by measuring the difference in the amount of free amine groups before and after the thiolation reaction using ortho-phthalicdialdehyde (OPA). The results of this study demonstrated a significantly higher degree of thiolation using OPA as compared to Ellman’s reagent. The authors explained it by assuming that some of the free sulfhydryl groups of thiolated gelatin might have undergone oxidation into disulfide, and since Ellman’s reagent could not detect disulfide, it might have resulted in underestimation. Accordingly, the authors suggested OPA as a superior reagent to determine the extent of protein thiolation. As expected, modified gelatin exhibited an increase in thiol content in proportion to the amount of AHTL used in the reaction. The reaction using 5-fold weight equivalents of AHTL showed the highest degree of gelatin thiolation of about ∼65%. Further to understand the effect of thiolation reagent on the degree of substitution, authors alternatively, also performed the reaction of gelatin B with 2-iminothiolane (Traut’s reagent) at 45^∘^ C for 45 min in phosphate buffer (pH 8). During this reaction, Traut’s reagent reacted with the ε-amine groups of the side chain of the basic amino acid residue (lysine) of the gelatin in a ring-opening reaction incorporating thiol groups. The determination of thiol content of modified gelatin by the OPA method revealed that Traut’s reagent was more efficient than AHTL for reacting with the free amine groups of proteins under identical reaction conditions. For instance, reaction using one weight equivalent of Traut’s reagent resulted in ∼70% thiolation of gelatin as compared to just ∼30% achieved using a similar weight equivalent of AHTL. Additionally, Traut’s reagent resulted in the thiolation of gelatin more rapidly (45 min) compared to AHTL (3 h). Based on these findings, the authors suggested Traut’s reagent as a better thiolation reagent for performing protein thiolation with a higher yield. In another interesting approach, Xu et al. reported the synthesis of thiolated gelatin by employing the carbodiimide coupling chemistry (Xu et al., 2020). Briefly, the authors mixed the aqueous solution of gelatin in distilled water with cysteamine and EDC in a molar ratio of 1:2:2 (carboxyl groups of gelatin/cysteamine/EDC). Subsequently, the pH of the reaction mixture was adjusted to ∼5, and the reaction was carried out at 37 °C for 4 h. Finally, the reaction was stopped by neutralizing the reaction mixture with NaOH, followed by purification of the product through dialysis and the determination of the degree of substitution using Ellman’s reagent. The results confirmed that the product contained a thiol content of 0.48 mmol/g, which corresponded to ∼39.2% of the degree of substitution. Of late, a number of studies have been reported in recent years wherein the thiolated gelatin has been synthesized following either of the above-described methodologies (Kommareddy and Amiji, 2005; Kommareddy and Amiji, 2007a; Rohrer et al., 2015; Pearce et al., 2021). For instance, Kommareddy synthesized the varying degree of thiolated gelatin by incubating gelatin B with 0.02, 0.04 and 0.1 weight equivalents of Traut’s reagent in plain deionised water (Kommareddy and Amiji, 2005; Kommareddy and Amiji, 2007a). The quantification of thiol groups in the modified gelatin through Ellman’sassay confirmed the increasing degree of thiolation with increasing weight equivalents of Traut’s reagent. Briefly, gelatin modified with 0.02, 0.04 and 0.1 weight equivalents of Traut’s reagent showed ∼6.08 mmol/g, ∼16.4 mmol/g and ∼43.7 mmol/g of free sulfhydryl groups, respectively, as compared to native gelatin (absence of sulfhydryl groups). Recently, in order to further increase the degree of thiolation, Duggan et al. employed a novel strategy of performing the thiolation in two steps (Duggan et al., 2015). In the first step, authors performed the amination of gelatin by introducing an amine group at the free carboxylic groups of its acidic amino acid residues through EDA-EDC coupling reaction in order to increase the overall number of free amine groups. In the second step, the aminated gelatin was used as a precursor for thiolation using Traut’s reagent. Notably, authors reported almost a ∼10-fold increase (663.2 ± 32.3 mol/g) in the thiolation of gelatin by using this two-step methodology in comparison to direct thiolation (60.2 ± 22.1 mol/g) without an amination step. Moreover, authors also optimised some of the crucial steps to prevent the oxidation of free sulfhydryl groups of the thiolated gelatin such as degassing of the reaction medium before the start of the reaction to remove oxygen or bringing down the pH of reaction mixture to acidic range (∼5) soon after the completion of reaction or performing the dialysis of the final product under acidic (∼5) environment. Currently, thiolated gelatin with the free sulfhydryl content of 150–350 μmol/g is commercially available from leading manufacturers (e.g., Sigma-Aldrich/Merck, etc.) of fine chemicals.

### 3.3 Acrylated gelatin

Methacrylate-and acrylate-functionalized gelatins have recently gained a lot of significance primarily due to their ability to undergo on-demand thiol- or photochemical-induced crosslinking under ambient conditions (aqueous environment, room temperature, neutral pH, etc.), yielding various kinds of structural organisation useful for drug delivery, tissue engineering and many other biomedical applications (Yue et al., 2015; Nagaraj et al., 2022). The functionalization of gelatin with methacrylate is performed through nucleophilic addition reaction between gelatin and methacrylic anhydride (MA) in which the methacrylic is added at amine (-NH_2_) and hydroxyl (-OH) groups containing side chain of gelatin, resulting in the conversion of amine group into methacrylamide group and hydroxyl group into methacrylate group (Yue et al., 2015; Nagaraj et al., 2022). However, the addition of methacrylic at the amine group predominates over its addition to hydroxyl groups, thereby resulting in the majority of methacrylamide groups and a minority of methacrylate groups in the modified gelatin (Yue et al., 2015; Nagaraj et al., 2022). Accordingly, methacrylate functionalized gelatin has recently been named gelatin methacryloyl (GelMA) by Yue and co-workers (Yue et al., 2015). There are numerous reports on the synthesis of GelMA (Yue et al., 2015; Nagaraj et al., 2022; Swanson et al., 2023; Van Den Bulcke et al., 2000; Aldana et al., 2019; Nichol et al., 2010). Briefly, this process involves dissolving gelatin (10% w/v) into deionised water or phosphate buffer (pH = ∼7) at 50^∘^C. To this, MA is added dropwise to reach the predetermined final concentration (% v/v). The reaction mixture is maintained at 50^∘^ C under constant stirring (240 rpm) for 2 h. Following this, the reaction mixture is diluted (typically 1:5) with preheated (50^∘^ C) phosphate-buffered saline (PBS) to stop the reaction, dialysed against water for 5–6 days for complete purification of the modified gelatin and freeze-dried to yield the GelMA in powder form (Yue et al., 2015; Nagaraj et al., 2022; Swanson et al., 2023; Van Den Bulcke et al., 2000; Aldana et al., 2019; Nichol et al., 2010). In this reaction, MA, being an organic compound, forms a dispersion upon its addition to the aqueous phase containing gelatin. Therefore, the rate of MA addition and the reactivity of the amine groups of gelatin are the two major factors influencing the degree of methacrylation. Generally, it has been seen that the slow addition of MA into the aqueous gelatin solution gives a higher degree of methacrylation. Similarly, a mild alkaline condition (pH ∼8) increases the reactivity of amine groups, thereby increasing the degree of methacrylation. The degree of methacrylation is usually determined by estimating the difference in the amount of free amine groups before and after the reaction using TNBS.The results from various studies have indicated that by increasing the amount of MA in the reaction mixture up to 20% (v/v), almost 80% of the free amine groups of the side chain of gelatin can be substituted with the methacrylamide group. Physicochemical characterisation of GelMA has confirmed that the addition of MA primarily happens at the ε-amino group of the lysine residues of gelatin, which accounts for only 5% of the total amino acid residues present in gelatin (Van Den Bulcke et al., 2000). Importantly, methacrylation does not influence the RGD sequence of gelatin, thereby retaining its mucoadhesive property. Furthermore, methacryloyl modification of gelatin does influence its thermo-reversible gelling property while increasing its gel strength and viscosity (Yue et al., 2015; Nagaraj et al., 2022; Swanson et al., 2023; Van Den Bulcke et al., 2000; Aldana et al., 2019; Nichol et al., 2010). On the other hand, depending on the gelatin type, degree of methacrylation and synthesis condition, the IEP of GelMA is significantly altered in comparison to the IEP of unmodified native gelatin (Yue et al., 2015; Nagaraj et al., 2022; Swanson et al., 2023; Van Den Bulcke et al., 2000; Aldana et al., 2019; Nichol et al., 2010). Of late, the acylate-functionalized gelatin has also been reported for various biomedical applications. The acylate-functionalized gelatin is synthesized following a similar synthetic strategy as that of GelMA by just replacing MA with acrylate anhydride in the reaction mixture (Yue et al., 2015). However, unlike GelMA, the acylate-functionalized gelatin does not facilitate rapid photo-induced crosslinking. Additionally, acylate-functionalized gelatin does not possess functional groups to support the mechanical strength and gel strength as that of GelMA (Yue et al., 2015). Moreover, the biocompatibility of acylate-functionalized gelatin is also poor as compared to GelMA. In view of the above-mentioned advantages, GelMA is a superior material to acylate-functionalized gelatin for biomedical applications. Like thiolated gelatin, the methacrylate- and acrylate-functionalized gelatins are also available from various commercial suppliers.

### 3.4 Pegylated gelatin

The PEGylation refers to attaching one or more molecules of polyethylene glycol (PEG) to the target material. The PEGylation of protein was reported way back in the 1970s by Davies and Abuchowsky, wherein the authors had reported the grafting of PEG on albumin and catalase proteins (Abuchowski et al., 1977a; Abuchowski et al., 1977b). Since then, the methodology of protein PEGylation has undergone significant advancements, with several chemical and enzymatic methods currently being employed for this purpose. These studies have revealed that PEG can be attached to a wide range of functional groups, such as amine, sulfhydryl, hydroxyl and amide groups, commonly available in the backbone of most of the proteins (Veronese and Pasut, 2005). Of these, PEGylation at the free amine group is widely preferred because of the abundance of free amine groups in the side chain of most of the proteins. Some of the PEG derivatives which are commonly used in the PEGylation of proteins at the free amine group are PEG-aldehyde, PEG-carboxylates (e.g., PEG–succinimidyl succinate, PEG–amino acid–succinimidyl ester, PEG–peptide–succinimidyl ester, etc.) and PEG-carbonates (e.g., PEG–p-nitrophenyl carbonate, PEG–benzotriazolyl carbonate, PEG–2,3,5-trichlorophenyl carbonate, PEG–succinimidyl carbonate, etc.) (Veronese and Pasut, 2005). Among these, PEG-aldehyde is attached to the amine group of proteins through the alkylation reaction and maintains the overall charge of the native protein even after PEGylation due to the formation of a secondary amine. Whereas, PEG-carboxylates and PEG-carbonates are attached to the amine group of proteins through acylation reaction, leading to the formation of an amide linkage between the amine group of protein and PEG. This type of reaction leads to loss of positive charges from the native protein following PEGylation (Veronese and Pasut, 2005). The PEGylation at free sulfhydryl groups is preferred primarily for proteins containing reduced cysteine residues. However, this strategy can also be employed by introducing either cysteine residues through genetic engineering or the free sulfhydryl groups by chemical modification in the target proteins (Veronese and Pasut, 2005). The thiol reactive PEG derivatives commonly employed for PEGylation of proteins at the free sulfhydryl sites are PEG–pyridildisulphide, PEG–maleimide, PEG–vinylsulfone and PEG–iodoacetamide (Veronese and Pasut, 2005). Further, the PEGylation of proteins at selective amino groups can also be achieved through a two-step chemical reaction in which the first step comprises the protection of a few functional groups in order to avoid the PEGylation at unwanted sites, followed by a second step of attaching PEG at the unprotected site (Veronese and Pasut, 2005). An alternative to chemical methods is the recently reported enzyme-catalysed procedures, which have been utilized to attach PEG specifically at the amide group of glutamines or at the hydroxyl group of serines and threonines in proteins (Veronese and Pasut, 2005). For instance, the enzyme transglutaminase performs the selective PEGylation at glutamine residues of protein by using amino-PEG as the nucleophilic donor (Veronese and Pasut, 2005). In this reaction, transglutaminase catalyses the transglutamination between amino-PEG and the amide group of glutamines, yielding selective PEGylation at glutamine residues of the proteins. Similarly, the enzyme-catalysed PEGylation of proteins at the hydroxyl group of serine and threonine residues is performed by using two sets of enzymes (Veronese and Pasut, 2005). In the first step, the target protein is selectively glycosylated by attaching N-acetylgalactosamine (GalNAc) at the hydroxyl group of serine and threonine residues with the help of O-GalNAc-transferase enzyme (Veronese and Pasut, 2005). In the second step, the glycosylated protein is used as the substrate for PEGylation catalysed by the enzyme sialyltransferase. In this reaction, sialyltransferase catalyses the addition of sialic acid-PEG to the O-GalNAc residues (Veronese and Pasut, 2005). Recently, PEGylation of gelatin has also emerged as an important strategy to improve its properties, such as biocompatibility and circulation time and also to reduce its immunogenicity (Abuchowski et al., 1977a; Abuchowski et al., 1977b; Veronese and Pasut, 2005). These criteria are an essential requirement to be present in a material like gelatin envisioned for biomedical applications. PEGylation of gelatin is mostly reported using chemical methods. For instance, Kim and Byun in the late 1990s reported the synthesis of PEGylated gelatin by attaching the monomethoxypolyethylene glycol (mPEG) at the amine groups of gelatin using carbodiimide coupling chemistry (Kim and Byun, 1999). In this study, the authors first chemically modified mPEG to introduce a carboxyl group at the terminal end. Subsequently, the carboxylate group of mono-carboxylated mPEG was activated using N,N′-dichlorohexylcarbodiimide (DCC) in the presence of hydroxysuccinimide (HOSU). The activated mPEG was then reacted with gelatin in an aqueous/methanol mixture for 2 h at room temperature. The product was purified by dialysis, freeze-dried and used for subsequent studies. The FTIR characterisation of the purified product confirmed the conjugation of mPEG with the amine groups of gelatin through amide linkage. Additionally, authors also determined the degree of PEGylation by measuring the amount of unreacted mPEG, which indeed confirmed that one molecule of gelatin was coupled with ∼205 molecules of mPEG. Subsequently, numerous studies have reported the synthesis of PEGylated gelatin for various biomedical applications. A few of them are discussed here. For instance, Kushibiki et al. recently prepared PEGylated gelatin by acylation reaction between the amine groups of gelatin and succinimidylsuccinate-methoxy PEG as the nucleophilic donor (Kushibiki et al., 2004). The reaction was carried out in DMSO under constant stirring for 3 h, and the modified gelatin was purified through dialysis. In order to study the effect of the molecular weight of PEG on the degree of PEGylation, the authors employed PEG of varying molecular weights (2–12 kDa) in the conjugation reaction. Additionally, authors also varied the amount of PEG to achieve varying degrees of PEGylation.The degree of PEGylation was examined by measuring the difference in the number of amine groups before and after the reaction. The findings of this study revealed that PEG was successfully grafted onto gelatin. The degree of PEGylation increased with an increase in the amount of PEG added initially in the reaction mixture. The reaction of PEG with gelatin in the 2:1 M ratio with respect to the molar concentration of amine groups of gelatin resulted in 100% PEGylation of all the free amine groups, irrespective of the molecular weight of PEG. Notably, this study also established that gelatin with 85%–100% of PEGylation exhibited a micellar structure with gelatin extending towards the core and PEG molecules covering the surface of the micelle. Further, the critical micellar concentration (CMC) of PEGylated gelatin depended on the molecular weight of PEG. Gelatin conjugated with a PEG of molecular weight of 2 kDa invariably showed a slightly lower CMC value than that conjugated with PEG of molecular weight of 12 kDa. Moreover, gelatin with 85%–100% of PEGylation exhibited a longer circulation half-life as compared to non-PEGylated gelatin. In another study, Cao et al. employed yet another strategy of synthesizing the PEGylated gelatin (Cao et al., 2020). The authors first introduced the free sulfhydryl groups at the free amine group of gelatin A (bloom strength:175) using Traut’s reagent and subsequently used this thiolated gelatin as a substrate to attach PEG by Michael-type addition reaction using 4-fold molar excess (with respect to molar concentration of free sulfhydryl groups in the thiolated gelatin) of poly(ethylene glycol) diacrylate (PEGDA, 8 kDa) as the thiol reactive nucleophilic donor. The authors reported ∼53% PEGylation efficiency with respect to the total number of freely available amine groups in the native gelatin through this methodology. In yet another strategy, Kaul and Amiji reported the synthesis of pegylated gelatin through a two-step reaction pathway (Kaul and Amiji, 2002). In the first step, authors converted the terminal hydroxyl group of PEG into epoxide through epoxidation reaction by incubating PEG with epichlorhydrin in the presence of triethanolamine. Subsequently, PEG epoxide was incubated with gelatin in alkaline borate buffer at 40 °C for 14 h, leading to grafting of PEG-epoxide to the primary amine groups through an addition reaction. The product was purified through dialysis followed by lyophilization and the extent of pegylation was determined by quantifying the amine groups before and after the conjugation reaction using the TNBS assay. The results indicated that the reaction of 0.5 g of PEG-epoxide per gram of gelatin resulted in about 30% PEGylation. Nevertheless, there have also been studies on the development of analytical methods for the accurate quantificationof the PEGylation of gelatin. In this context, recently Zillies et al. reported the potential of the combination of asymmetrical flow field-flow fractionation (AF4) and refractive index (RI) detection for the quantitative measurement of the degree of PEGylation (Zillies et al., 2007). The novelty of this methodology lies in the quantification of PEGylation of gelatin in the reaction medium itself without any further processing. In order to do so, the methodology relies on the separation of unreacted PEG from its reaction partner using AF4. During the AF4, the undissolved, suspended and dissolved samples of a wide molecular mass (∼10^4^–10^18^ Da) or size (1 nm - 100 µm) range are concurrently separated as layers of different velocities due to the application of cross flow or the field of separation perpendicular to the direction of laminar carrier flow in the separation channel. The authors have successfully shown the separation of free or unreacted PEG from PEGylated gelatin nanoparticles in the reaction medium by applying AF4 (Kaul and Amiji, 2002). Briefly, gelatin nanoparticles were prepared by crosslinking gelatin with glutaraldehyde, and PEG was attached to the residual aldehyde groups present on the surface of gelatin nanoparticles using amino-mPEG as the nucleophilic donor. The reaction was carried out in aqueous medium containing gelatin nanoparticles (20 mg/mL) and the varying concentrations of amino-mPEG (0.125, 0.25 and 1.0 mg/mg of nanoparticles) at 25^∘^ C for 2 h. Subsequently, the amount of unreacted PEG in the reaction medium was determined by performing AF4 followed by RI detection and using a calibration curve prepared using RI of the known concentration of PEG. Through this analysis, the authors could determine a PEGylation of 0.35 mg of PEG/mg of gelatin nanoparticles at the lowest used concentration of PEG in the reaction mixture, and it represented ∼100% attachment of PEG molecules to gelatin nanoparticles. The higher concentration of PEG in the reaction mixture did not show any improvement in the PEGylation efficiency. Overall above studies together suggest that PEGylation of gelatin can be performed in aqueous as well as organic media by reacting gelatin directly with the commercially available PEG derivatives or by chemically incorporating specific functional groups in gelatin or PEG, which can facilitate their conjugation with each other. The degree of PEGylation is mainly influenced by the conjugation strategy, which in turn governs the physicochemical property. Additionally, the molecular size of PEG can also influence the physicochemical properties of gelatin.

### 3.5 Azido-functionalized gelatin

Among gelatin derivatives, azido-functionalized gelatin has also received significant attention because of its ability to undergo alkyne-azide cycloaddition (also known as click coupling) as the cross-linking reaction in the presence of alkyne-containing substrates and forming the gel-like network (Avti et al., 2013; Piluso et al., 2018). The azido-functionalization of a protein is commonly performed by diazo transfer through the reaction of an electrophilic, azido-functional small molecule with the nucleophilic free amine group of lysine residues in its backbone (Sugawara and Matsuda, 1995). Over the years, quite a few works have been done on the optimisation of reaction conditions for achieving the efficient functionalization of gelatin with an azido group. For instance, Matsuda and Sugawara, during the 1990s, prepared the phenyl azido-derivatives of gelatin for surface coating application (Sugawara and Matsuda, 1995). The synthetic strategy of this study involved adding N-(4-azidobenzoyloxy) succinimide to the aqueous buffered (phosphate buffer, pH7.4) solution of gelatin under constant stirring and incubating the reaction mixture at 4^∘^ C for 24 h. During this process, an activated ester group can specifically react with the primary amine group of gelatin, resulting in the derivatization with the phenyl azido group. Authors indeed confirmed the derivatization of gelatin by recording the characteristic IR absorption peak of the azido group at around 2,130 cm^-1^. Almost during same time, Narayani and Rao reported the synthesis of gelatin azide by following the stepwise conversion of the carboxylate groups of acidic residues (aspartic and glutamic acid) of gelatin into azide groups (Narayani and Rao, 1996). Briefly, the reaction involved incubating gelatin with an excess of methanol in the presence of 0.1 NHCl for 1 week in order to facilitate the formation of methoxy ester (methylation reaction) of gelatin. Subsequently, gelatin easter was reacted with hydrazine hydrate (2% w/v) at room temperature for overnight to yield gelatin hydrazides. Following this, gelatin hydrazide was converted to gelatin azide through a diazotization reaction in the presence of acid (0.6 N HCl) and nitrite (5% sodium nitrite). Authors confirmed the conversion of carboxylic groups of gelatin into azide groups through FTIR characterisation. Further, the degree of substitution was determined in terms of the conjugation efficiency of gelatin azide with methotrexate (MTX). This analysis suggested about ∼203 µg of MTX attached to each mg of gelatin azide, as compared to negligible conjugation of MTX with native gelatin. Together, these findings established the efficient functionalization of gelatin with an azido group. In a recent study, Truong et al., reported the functionalization of gelatin with o-nitrobenzyl (NB) azide using a two-step polymer-analogous reaction involving carbodiimide coupling (Truong et al., 2015). Briefly, the authors used gelatin and NB-azide acid as the precursors. In the first step, the carboxylic group of NB-azide acid was activated using EDC/HoBt in DMF, and subsequently, the activated NB-azide acid was reacted with gelatin to yield NB-azide-functionalized gelatin. The basic principle behind the attachment of NB-azide acid to gelatin was the formation of an amide linkage between the carboxylic group of activated NB-azide and the amine group of the lysine residue of the gelatin. Here, the major challenge for the authors was to optimise the reaction medium for dissolving the reactants, as gelatin is insoluble in most solvents except water, whereas NB-azide, due to its hydrophobicity, is barely soluble in water. The authors found that a 3:2 (v/v) mixture of DMF and water was most suitable for dissolving both gelatin and the activated NB-azide acid. Further, to determine the degree of conjugation, the product was purified through dialysis (against water), and the concentration of primary amine was measured by fluoraldehyde assay using lysine as the standard. The difference in the concentration of primary amine groups before and after the functionalization reaction was used to calculate the degree of substitution. This analysis revealed that reacting NB-azide with gelatin in a molar ratio greater than 0.1:1 resulted in the extremely poor recovery of modified gelatin due to its precipitation during dialysis. The precipitation of the modified gelatin was due to the increase in hydrophobicity following conjugation with NB-azide. Notably, authors reported a maximum conjugation efficiency of 5% (with respect to total primary amine groups available in the gelatin) in the water recoverable form of modified gelatin. In another similar study, four types of azide-modified gelatin with increasing degrees of substitution were synthesized by reacting 25, 50, 75 and 100 M equivalents of amine-reactive azide-PEG4-NHS ester to 1 mol equivalent of primary amine groups of gelatin in the buffered aqueous medium (pH ∼7) at 37 °C for 2 h (Daniel et al., 2014). The azide-modified gelatin was recovered from the reaction mixture through dialysis, with the yield ranging from 71%–82%. The degree of azide modification was estimated from the amount of remaining reactive amine groups in the modified gelatin by a fluorescamine assay in comparison to native unmodified gelatin. This assay indicated that about ∼37%, ∼67%, ∼87% and ∼98% of reactive amine groups (mol%) in gelatin were modified with the azido group at the increasing molar ratios of 25, 50, 75 and 100 of amine-reactive azide-PEG4-NHS ester, respectively. Alternatively, researchers have also performed the azido functionalization of gelatin by coupling the carboxylic group of its acidic amino acid residues with the azido-functional small molecule containing a free amine group, such as azido-PEG3-amine and 4-azidoaniline (Testore et al., 2025; Heo et al., 2016). Briefly, such reactions involved the activation of the carboxylic groups of gelatin with EDC/NHS or HoBt in an aqueous buffered medium, followed by nucleophilic attack by azido-amine compounds (1 mol per mole of carboxylic groups of gelatin), resulting in the formation of azido-conjugated gelatin derivative. The results indicated that a maximum of about ∼27% substitution could be achieved under the optimised conditions of the above methodology. Lately, these synthetic strategies have been employed by multiple research groups either as such or with some modifications to prepare azido-functionalized gelatin. One of the limitations of these strategies is the insertion of azido groups primarily in the side chain of basic or acidic residues of the gelatin, resulting in the extension of the side chain and, in turn, the hydrophobicity of modified gelatin. In order to overcome this, recently, Keller et al. introduced a novel strategy of synthesizing azido-modified gelatin by using the azido transfer reagent imidazole-1-sulfonyl azide hydrochloride (Keller et al., 2021). This reagent offered the one-step synthesis of azido-modified gelatin in an aqueous environment with minimal side chain extension. The reaction condition comprised mixing 0.8 M equivalent of imidazole-1-sulfonyl azide hydrochloride to per molar equivalent of lysine residues of gelatin in water in the presence of 8.5 and 0.6 M equivalents of potassium carbonate and copper sulphate, respectively, at 37^∘^ C for overnight under constant stirring, followed by dialysis and freeze drying to yield the azido-modified gelatin as the powder. The azido modification of gelatin was confirmed by the appearance of a new characteristic absorption band at 2,102 cm^−1^ in the FTIR spectrum and by the decrease of signal intensity (at 3.0 ppm) corresponding to ε-CH_2_ group of the lysine residues with concurrent appearance of a new signal around 3.3 ppm in the ^1^H-NMR spectra of modified gelatin. The quantification of the amount of primary amine groups (by TNBS assay and ^1^H-NMR spectroscopy) before and after the diazo transfer reaction suggested that almost 80%–90% of the amino groups were modified with azido groups. Further, authors also reported that azido modification of gelatin significantly altered its gelling property and IEP. For instance, the azido-modified gelatin exhibited the *T*_gel, down_ (gel transition temperature measured by lowering the temperature from 40 to 10^∘^ C) and *T*_gel,up_ (gel transition temperature measured by increasing the temperaturefrom 10 to 40^∘^ C) of 18.6^∘^ C and 25.3^∘^ C respectively, as compared to 24.7^∘^ C and 31.4^∘^ C respectively, of native unmodified gelatin. Similarly, the IEP of gelatin also decreased from 4.9 to 4.2 after azido modification. Overall, this study optimised a simple synthetic mechanism for the preparation of azido-gelatin. As of today, the ever-increasing demand for azido-modified gelatin for biomedical applications has driven its commercial availability from multiple manufacturers.

### 3.6 Selenogelatin

The idea of derivatizing gelatin with selenium was recently conceived by our group, considering the growing importance of selenium in the field of healthcare applications (Rayman, 2000; Rayman, 2012; Jain and Priyadarsini, 2017; Papp et al., 2007; Gallo-Rodriguez and Rodriguez, 2024; Chuai et al., 2021). For instance, selenium, once regarded as a toxic substance, has now emerged as an important micronutrient required for maintaining normal physiological functions in animals and humans (Rayman, 2000; Rayman, 2012; Jain and Priyadarsini, 2017; Papp et al., 2007). Some of the key physiological functions imparted by selenium include antioxidant defence against oxidative stress, thyroid metabolism, fertility and immune enhancement through its incorporation in the form of selenocysteine (21st amino acid) into a class of proteins called selenoproteins (Papp et al., 2007). A total of 25 selenoproteins have been identified to date in case of humans of which mostly are the oxidoreductase type of enzymes and contain selenocysteine residue in their active site (Papp et al., 2007). This clearly suggests that selenium mainly plays the catalytic role for facilitating redox reactions, which is attributed to its higher polarizability or ability to easily donate electrons (Jain and Priyadarsini, 2017). Glutathione peroxidase, thioredoxin reductase, and iodothyronine deiodinase are some of the examples of oxidoreductase enzymes containing selenocysteine in their active site (Jain and Priyadarsini, 2017; Papp et al., 2007). Moreover, decades of research have established that selenium deficiency leads to the onset of various diseases, whereas its consumption beyond a threshold level can cause life-threatening toxicity (Rayman, 2000; Rayman, 2012; Jain and Priyadarsini, 2017; Papp et al., 2007). As per the WHO guideline, the recommended daily allowance and the upper threshold limit for daily intake of selenium in the elemental form are 35–50 µg and 400 μg, respectively (Rayman, 2000; Rayman, 2012; Jain and Priyadarsini, 2017; Papp et al., 2007). Additionally, selenium in the various chemical forms has also been shown to exhibit a wide range of pharmacological activities (Jain and Priyadarsini, 2017; Gallo-Rodriguez and Rodriguez, 2024; Chuai et al., 2021). All these considerations prompted our group to anticipate that selenium-derivatized gelatin may be useful for future applications as a dietary source of selenium as well as for pharmacological applications. In order to address this hypothesis, our group recently reported a novel synthetic strategy of attaching a diselenide moiety to the free amine group of the lysine residues of gelatin using 3,3′-diselenodipropionic acid (DSePA) as the diselenide transfer reagent (Das et al., 2023; Das et al., 2024). The benefits of using DSePA as a selenium transfer reagent are its water solubility, amicability to undergo carbodiimide coupling, and the relatively shorter carbon chain length, thus facilitating the recovery of modified gelatin in the water-soluble form with minimal side chain extension or hydrophobicity. The methodology in brief comprised activating the carboxylic group of a predetermined concentration of DSePA using 4 equivalent of NHS and 2 equivalent of EDC for 2 h in water at room temperature followed by the addition of the aqueous solution of gelatin at a final concentration of 1% w/v and continued stirring for pre-determined time points at room temperature to form the amide linkage between the carboxylic group of DSePA and the free amine group of the lysine residues of gelatin. Notably, the molar ratio of DSePA to the primary amine groups of gelatin was chosen in such a way to ensure the maximum conjugation efficiency (Das et al., 2023). Further, to optimise the reaction time for maximum conjugation efficiency, the reaction between the activated DSePA and gelatin was terminated at pre-determined time points ranging from 2 to 24 h. The modified gelatin from the reaction mixture was purified by dialysis and freeze-dried for subsequent use. The conjugation of DSePA to gelatin was confirmed by the reinforcement of characteristic absorption bands of amide I, amide II, and amide III, a decrease in wavenumber of amide A and amide B bands and the disappearance of OH-stretching bands of DSePA in the FTIR spectrum of modified gelatin as compared to the native gelatin and neat DSePA. Additionally, the modified gelatin also showed the presence of bands corresponding to the ionisation energy level of selenium element at 52.5eV and 55.3eV (Se3d_3/2_ energy level) through X-ray photoelectron spectroscopy (XPS), confirming the successful functionalization of gelatin with selenium. The yield of reaction was calculated by taking into account the initial amount of protein added to the reaction mixture and the amount of modified protein recovered after the dialysis. This analysis indicated recovery of the modified gelatin with 80%–90% yield. Further, to calculate the conjugation efficiency, the amount of selenium in the modified gelatin was estimated through atomic absorption spectroscopy (AAS) and expressed as loading of selenium per mg of gelatin. Interestingly, conjugation efficiency or selenium loading into gelatin increased with increasing reaction time up to 8 h, whereas the subsequent increase of reaction time decreased the conjugation efficiency. The decrease in conjugation efficiency by the longer reaction time was attributed to the slow degradation of the diselenide bond during the activation of DSePA in the presence of the NHS. A maximum conjugation of ∼5 µg selenium/mg of gelatin was achieved after 8 h of reaction time. Having optimised the synthetic strategy of selenium functionalized gelatin, our group is currently exploring it for various biomedical applications such as wound healing, preparation of redox responsive nanoparticles and as a therapeutic agent against cancer (Das et al., 2023; Das et al., 2024). On a similar line, Pérez-araluce and co-workers have recently reported another simple one-step synthetic strategy for functionalizing gelatin with selenium (Pérez-Araluce et al., 2024). Briefly, the group performed the reaction by directly incubating gelatin (10% w/v) with 754 mg of selenium dioxide in phosphate-buffered saline for 4 h and purified the modified gelatin through dialysis. The authors proposed that the reaction proceeds with the conversion of selenium dioxide to selenous acid under an aqueous environment, followed by its addition to free amine groups of the lysine residues of gelatin. The incorporation of selenium in the gelatin was confirmed by acquiring the Se^77^-NMR, which showed the characteristic peak of selenium at 1309 ppm. Additionally, the ^1^H-NMR spectrum of modified gelatin also showed discernible differences as compared to native unmodified gelatin, supporting successful incorporation of selenium into gelatin. The estimation of selenium in the modified gelatin by AAS suggested a maximum loading of 2.3 ± 0.3 mg of selenium per of gelatin. Together, the findings of the above studies are expected to stimulate research on selenium-functionalized gelatin.

## 4 Biodegradation of gelatin derivatives

The biodegradation of gelatin and its chemically modified derivatives have been studied mainly in the context of drug delivery system or their ability to facilitate the release of entrapped payload (Figure 4). Generally, the bio-degradation of gelatin occurs through the hydrolysis mediated by the proteolytic enzymes specifically the MMP2 or gelatinase. Several recent studies have established that chemically modified derivatives of gelatin although can be fully bio-degraded, undergo slower proteolytic degradation compared to the native gelatin, a crucial property required for fabricating drug delivery systems to achieve the sustained release of payload for a long duration. Further, the kinetics of the degradation of chemically modified derivatives of gelatin depends on the degree of substitution. Generally, the kinetics of the proteolytic degradation of a gelatin derivative becomes slower with the increasing degree of substitution. For instance, Luo and Lai (2019), recently studied the role of gelatin amination on its resistance for biodegradation with an aim to design a long-acting injectable depot system for intracameral (ophthalmic) application (Luo and Lai, 2019). The authors prepared thermo-gels by grafting carboxyl-terminated poly(N-isopropylacrylamide) over aminated gelatin of the varying degree of substitutions (35.0 ± 1.7, 50.1 ± 2.6, 65.9 ± 3.2, and 99.8 ± 2.5 amine groups per molecule). The thermo-gels were loaded with pilocarpine and their biodegradation as well as the release behaviors were studied under physiological condition in the presence MMP2. The results clearly indicated that the increasing degree of amination increased the resistance of gelatin against biodegradation (enzymatic degradability) facilitating the extended-release profiles of pilocarpine and higher therapeutic efficacy. Simultaneously, these studies also indicated that the higher degree of amination may compromise the biocompatibility of gelatin through their accumulation within cells and by promoting the oxidative stress signals. In another study, Morimoto et al. fabricated cationic microshpheres (CGMS) using aminated gelatin and compared its drug release behavior with that of microshpheres (NGMS) based on a native gelatin (Morimoto et al., 2008). The authors studied the release of three model acidic peptide/protein drugs such as recombinant human insulin, bovine milk lactoalbumin and bovine serum albumin from CGMS and NGMS in a plain aqueous buffer medium without using any stimulus or biodegrading enzyme. The results indicated the slow release of protein drugs from CGMS as compared to NGSM. The authors explained this observation by postulating that the release of protein drugs from CGMS and NGMS was governed by the weaking of electrostatic interaction between the drugs and carrier during the initial phase of release kinetics and by the hydrolysis of the carrier during the extended period of release kinetics. Since CGMS exhibited slower release, it indirectly suggested the slower hydrolysis of aminated gelatin as compared to native gelatin. Together above studies establish that amination reduces the bio-degradability of gelatin. With respect to thiolated gelatin, it exhibits biodegradability similar to that of native gelatin. Notably, the thiolated gelatin apart from undergoing proteolytic degradation can also degrade through redox processes involving cellular reducing agents such as (GSH, NADH and NADPH). For instance, extensive studies by Mansoor Amiji and his group has shown that protein nanoparticles based on thiolated gelatin facilitate the significantly higher release of payload under reducing environment compared to non-reducing environment due to the accelerated hydrolysis of thiolated gelatin by reducing agents (Kommareddy and Amiji, 2005; Kommareddy and Amiji, 2007b; Xu et al., 2014). Similarly, our group has reported the significantly higher release of payload from the nanoparticles of selenium functionalized gelatin under reducing environments suggesting their susceptibility for undergoing reductive bio-degradation (Das et al., 2024). Indeed, the reductive bio-degradation of selenogelatin is expected to higher than thiolated gelatin due to be the higher lability of diselenide linkage (172 kJ/mol) as compared to the disulfide linkage (240 kJ/mol). Further, gelatin methacryloyl (GelMA) undergoes slow degradation as compared to native gelatin and this property has enabled its wide spread use for the tissue engineering applications (Heltmann-Meyer et al., 2021). One of the criterions for a biomaterial to be suitable for tissue engineering application is its long-term stability and ability to promote vascularization. In this context, a recent study examined the in vivo degradability of GelMA-based hydrogel using rat arteriovenous loop (AVL) model. Briefly, rat AVLs embedded with GelMA hydrogel was implanted in the groin and its degradation was monitored in terms of the increase in the level of degrading enzyme like MMP3. The results indicated that GelMA hydrogels were stable for at least 4 weeks in the rat AVL model without compromising with biocompatibility. Moreover, GelMA hydrogels in the AVL model promoted vascularization and the formation of connective tissue. Together these results confirmed the slower biodegradability of GelMA. Another similar study examined the effect of PEGylation on the degradation rate of gelatin-PEG composite hydrogel (Zhou et al., 2016). In this study, gelatin-PEG composite hydrogel was subjected to enzymatic degradation in aqueous buffer containing collagenase. The degradation of gel was quantified in terms of weight loss and the correlation of the concentration of gelatin or the concentration of PEG or the concentration of crosslinking agent (glutaraldehyde) with the degradation rate was established. The results indicated that the concentration of crosslinking agent was the most effective factor followed by the concentration of PEG which had an antagonistic effect on the degradation rate of composite hydrogel. This clearly suggested that the degree of PEGylation slows the bio-degradation of gelatin.

**FIGURE 4:**
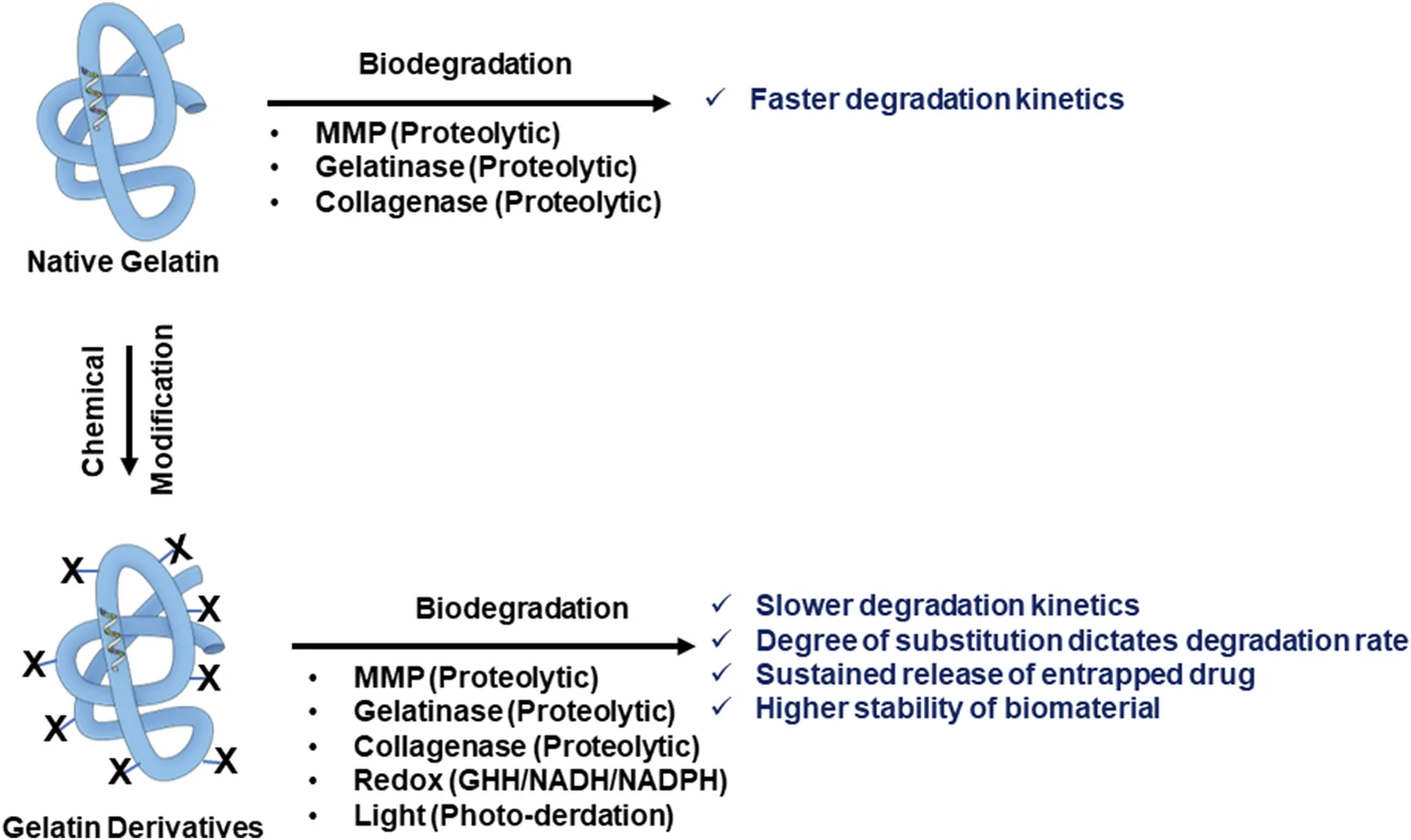
Biodegradation behaviour of native versues chemically modified gelatin.

In a yet another recent study, Zhao et al. prepared a series of hybrid hydrogels of PEG crosslinked with gelatin and examined their degradation for the controlled drug delivery applications (Zhao et al., 2024). The degradation of hydrogels was monitored by measuring their mass loss over time in plain aqueous buffer. The results indicated that the synthesized gelatin-PEG hydrogels were degradable, and the complete degradation time varied between 12 and 72 h depending on the degree of crosslinking. The gelatin with the higher degree of PEG crosslinking showed prolong degradation and drug release times. Further, delivery system based on azide functionalized gelatin have shown biodegradability similar to that of native gelatin. However, azide functionalization followed by crosslinking with PEG can impart additional feature of photo-degradability to the delivery system (Tamura et al., 2015; Daniel et al., 2014). The crosslinking of azide functionalized gelatin with PEG facilitates its photo-degradation through the creation of a photolabile, nitrobenzyl ether moiety. Overall, the bio-degradation of gelatin can be tuned to favor the drug delivery applications through various chemical modifications.

## 5 Mechanistic details on drug delivery and biomedical applications of gelatin derivatives

There are several dedicated review articles on the wide-ranging biomedical applications of gelatin, including its modified derivatives (Su and Wang, 2015; Milano et al., 2023; Zhang and Wang, 2024; Mohanto et al., 2023; Elzoghby, 2013; Yue et al., 2015; Bello et al., 2020; Jia et al., 2024; Cao et al., 2024). Accordingly, the present section purposefully provides only a brief overview of some of the recent developments in this field. Although gelatin and its modified derivatives can be engineered into various structural forms, including but not limited to hydrogel, capsule, microspheres, nanofiber and nanoparticle, it is the hydrogel which has received maximum attention for drug delivery and biomedical applications (Zhang and Wang, 2024; Mohanto et al., 2023; Elzoghby, 2013; Yue et al., 2015). The basic principle behind the formation of gelatin-based hydrogel is the self-assembly, primarily through crosslinking, wherein individual gelatin molecules are covalently linked with each other with the help of a crosslinking agent to form a dense network-like structure. Glutaraldehyde is one of the widely used cross-linking agents in the preparation of gelatin-based hydrogel (Zhang and Wang, 2024; Bello et al., 2020; Olde Damink et al., 1995; Talebian et al., 2007). The glutaraldehyde establishes covalent linking between free amine groups of lysine residues or the free hydroxyl groups of hydroxproline residues of gelatin using its bifunctional aldehyde groups through Schiffbase formation (Olde Damink et al., 1995; Talebian et al., 2007; Mugnaini et al., 2023; Farris et al., 2010). Although glutaraldehyde-mediated crosslinking is an efficient process of making gelatin hydrogel, the limited availability of lysine residue in the backbone of gelatin results in poor mechanical stability of the resulting hydrogel (Olde Damink et al., 1995; Talebian et al., 2007; Mugnaini et al., 2023). However, the advent of various types of chemically modified gelatin has revolutionised the hydrogel technology by not only facilitating the remarkable improvements in the rheological and mechanical properties but also imparting tunable stimuli responsiveness behaviour to the hydrogel. For instance, an amide derivative of gelatin has a higher number of freely available amine groups than native gelatin. Accordingly, it undergoes more efficient and rapid crosslinking than native gelatin in the presence of glutaraldehyde (Talebian et al., 2007; Mugnaini et al., 2023; Farris et al., 2010). Additionally, the mechanical strength of amido-gelatin hydrogel is much superior to that of native gelatin. Similarly, the aqueous solution of azido derivative of gelatin permits the rapid hydrogel formation through the bio-orthogonal click reaction in the presence of a crosslinking reagent having dibenzocyclooctyne (DBCO) as the reactive moiety (Truong et al., 2015; Testore et al., 2025; Daniel et al., 2014; Watanabe et al., 2020; Contessi Negrini et al., 2021). During this reaction, the DBCO group of the crosslinking agent undergoes a click reaction with the azide group of gelatin, resulting in the formation of a hydrogel. Alternatively, an aqueous solution of GelMA facilitates rapid hydrogel formation through photo-crosslinking. Briefly, the aqueous solution containing GelMA and photo-initiator (such as irgacure 2,959 or lithium phenyl-2,4,6-trimethyl benzoyl phosphinate) upon exposure to UV light forms the hydrogel (Yue et al., 2015; Nagaraj et al., 2022; Swanson et al., 2023; Van Den Bulcke et al., 2000; Aldana et al., 2019; Nichol et al., 2010). During this process, the photo-initiator facilitates the polymerisation of methacrylamide moieties of gelatin through radical reaction, leading to the formation of a cross-linked hydrogel. Another important derivative, thiolated gelatin, permits dithiothreitol (DTT) mediated crosslinking to form a hydrogel (Gyarmati et al., 2013; Van Vlierberghe et al., 2011; Van Hoorick et al., 2021; Watanabe et al., 2011). Like glutaraldehyde, DTT is another widely used reagent for protein crosslinking. It allows crosslinking of protein through the formation of disulfide linkages between the free sulfhydryl groups of cysteine residues using its bifunctional sulfhydryl groups. However, due to the absence of cysteine residues in native gelatin, DTT cannot be utilized for its crosslinking. This limitation can be overcome by using thiolated gelatin (Gyarmati et al., 2013; Van Vlierberghe et al., 2011; Van Hoorick et al., 2021; Watanabe et al., 2011). Further, the major advantage of using the above-discussed derivatives of gelatin is their commercial availability with varying degrees of substitution, which permits researchers to fabricate hydrogels of desired rheological and mechanical parameters. Apart from hydrogel, gelatin nanoparticles constitute another major structural form for biomedical applications (Su and Wang, 2015; Milano et al., 2023; Zhang and Wang, 2024; Elzoghby, 2013). Gelatin nanoparticles can be prepared using various strategies, including but not limited to nanoprecipitation, desolvation, coacervation, polyelectrolyte complexation, emulsification, electrospray and spray-dry. For more details about these techniques, authors can refer to previous comprehensive reviews on the subject (Zhang and Wang, 2024; Elzoghby, 2013; Madkhali, 2023). The foremost biomedical application of gelatin-based nanoparticle is to act as the carrier or delivery system for drug molecules to enhance their bioavailability (Zhang and Wang, 2024; Elzoghby, 2013; Madkhali, 2023). In this regard, one of the requirements of the drug delivery system is to escape the removal from circulation by RES. In order to achieve this, pegylated gelatin is preferred over other derivatives for fabricating a nanocarrier because of its ability to evade the recognition by RES and, in turn, to enhance the circulation half-life of entrapped molecules. Briefly, optimum PEGylation provides hydrophilic shielding to gelatin-based nanoparticles, which allows them to escape the opsonisation and, in turn, clearance from the circulation (Kommareddy and Amiji, 2007a; Kushibiki et al., 2004; Santoro et al., 2014). Another important requirement of a drug delivery system is to facilitate higher cellular uptake as well as the controlled-on-demand release of entrapped drug molecules at the target site. The cellular uptake of drug delivery system specially nanoparticles is governed by their physico-chemical properties such as size, shape, surface charge and rigidity (Das et al., 2019; Augustine et al., 2020). Among these parameters, size, shape and surface charge of gelatin nanocarriers are dictataed by the method of their preparation rather than the chemical nature of gelatin. Alternatively, rigidity of nanocarriers can be effectively controlled by varying the degree of chemical modification/cross linking of native gelatin (Yildirim et al., 2023; Das et al., 2026). Recent studies have shown that gelatin nanoparticles with higher degree of chemical crosslinking exhibit higher cellular uptake by favoring the endocytosis process (Yildirim et al., 2023; Das et al., 2026). Accordingly, chemically modified gelatin is preferred over native gelatin to achieve higher cellular uptake by controlling the rigidity/mechanical properties of the nanocarriers. Further, gelatin-based drug delivery systems generally facilitate the targeted release of the payload to the cancerous cells due to the interaction of their RGD tripeptide moiety (present in their studture) with the intgrin recepotors overexpressed on tumor cells (Morán et al., 2018; Lu et al., 2020). Additionally, gelatin-based carriers can also undergo selective degradation at tumor site by matrix metalloproteinase (MMP) to facilitate the targeted release of the payoload (Vaghasiya et al., 2021). Furthermore, thiolated gelatin offers redox responsiveness to the hydrogel/nanocarrier because of the susceptibility of the disulfide linkages to cleavage by reducing agents (Kommareddy and Amiji, 2005; Kommareddy and Amiji, 2007b; Xu et al., 2014). Thus, hydrogel/nanocarriers prepared using thiolated gelatin selectively release the entrapped drug molecule in cells having elevated levels of reducing redox equivalents like glutathione (GSH). This kind of redox-responsive drug delivery systems are highly useful for improving the selectivity of anticancer drugs towards cancerous cells, as these cells are known to have elevated GSH levels as compared to non-cancerous cells (Kommareddy and Amiji, 2005; Kommareddy and Amiji, 2007b; Xu et al., 2014). On a similar line, diselenide-functional gelatin also offers redox-responsive release of entrapped drug by undergoing cleavage of the diselenide bond under the oxidizing and/or reducing environment (Das et al., 2024). Moreover, our group has recently shown that diselenide functionalized gelatin (R-Se-Se-R) accelerates wound healing through a novel mechanism of nitric oxide (NO) generation. Briefly the diselenide functonalized gelatin (R-Se-Se-R) catalyses the reduction of exogenously added NO donor (RSNO) such as S-nitroso-N-acetylpenicillamine (SNAP) through glutathione peroxidase-like activity leading to on-site production of NO (Das et al., 2023). The mechanistic investigation on the catalytic cycle of the diselenide functonalized gelatin has indicated that it can directly react with RSNO producing NO and R-Se-S-R intermediate. The R-Se-S-R intermediate is recycled back to R-Se-Se-R through recomination reaction. Alternatively, diselenide functonalized gelatin (R-Se-Se-R) can react with endogenously available glutathione (GSH) leading to formation of RSeSG which can subsequently react with RSNO to produce NO and R-Se-S-R. The R-Se-S-R is again recycled back to R-Se-Se-R to continue the catalytic cycle.

In addition to above benefits, the summary of some of the recent studies (last 5 years) on the biomedical applications of gelatin derivatives is given in Table 2 and Figure 5.

**TABLE 2:** Biomedical application of chemically modified gelatin demonstrated using pre-clinical model*s*.

Formulation	Chemical modification	Intended application	Key findings	Ref
Ag-nanoparticle entrapped gelatin methacryloyl (GelMA) soft gel	Methacryloyl	Tissue engineering & wound healing	15% GelMA gel loaded with ∼10 nm AgNPs exhibited slow and sustained release of nanoparticles over a duration of 72 h. Under simulated condition, AgNPs-GelMA gel supported accelerated wound healing by minimizing microbial infections against Gram-positive and Gram-negative bacteria and enabling faster fibroblast (NIH3T3) migration	(Jahan et al., 2019)
Calcium phosphate-gelatin methacryloyl (CaPs/GelMA) composite nanofibers	Methacryloyl	Bone tissue engineering	CaPs/GelMA composite nanofibers exhibited periosteum mimicking activity under *in vitro* conditions. The nanofibers also showed favourable biocompatibility, angiogenesis and osteogenesis during co-culture with HUVECs and MC3T3-E1 cells	(Liu et al., 2020)
Decellularized liver matrix-gelatin methacryloyl (DLM-GelMA) composite scaffold	Methacryloyl	Hepatic encephalopathy	A bioartificial liver support system fabricated by integrating GelMA with DLM and hepatocytes reduced ammonia (toxin) concentration by 45%, thus mimicking the detoxification function of whole liver	(Wu et al., 2020)
Gelatin methacryloyl (GelMA) wearable tactile sensors	Methacryloyl	Biosensing	The designed GelMA tactile sensor showed high-pressure sensitivity of 0.19 kPa^−1^ and one order of magnitude lower limit of detection (0.1 Pa) compared to other hydrogel pressure sensors due to its excellent mechanical and electrical properties (dielectric constant). Durability up to 3,000 test cycles	(Li et al., 2020)
Alginate dialdehyde-gelatin (ADA-GEL) hydrogel nanocomposites	Amination followed by conjugation with alginate	Bone tissue engineering & dug (Icariin) delivery	ADA-Gel containing icariin loaded mesoporous silica-calcia nanoparticles induced osteoblast proliferation, adhesion, and differentiation. Moreover, it improved stiffness and confered bioactivity in simulated body fluid by favourably modulating icariin release profile and kinetics	(Monavari et al., 2021)
Thiol-norbornene functionalized gelatin hydrogels	Thiolation	3D printing	The thiol-norbornene functionalized gelatin hydrogel exhibited faster photocrosslinking and superior biocompatibility and processability	(Van Hoorick et al., 2021)
Dopamine grafted gelatin (GelDA) hydrogel	Amination using dopamine	Tissue adhesive, wound dressing & wearable devices	A self-healing hydrogel prepared by incorporating graphene oxide (GO) into GelDA hydrogel functioned as the adhesive electrodes for EMG monitor and successfully captured electromyography (EMG) signals of finger movement. The incorporation of GO improved the electrical and mechanical properties of GelDA hydrogel	(Han et al., 2021)
Cross-linked elastin in gelatin/PEG hydrogel	PEGylation	Tissue engineering	Gelatin–PEG-elastin hydrogel supported thet viability and promoted the proliferation of encapsulated fibroblast cells. Moreover, the hydyogel induced the expression of collagen alpha-1 and elastin from the encapsulated fibroblast thus justifying its potential for wound healing application	(Cao et al., 2020)
Gelatin methacryloyl (GelMA)-AuNP scaffolds	Methacryloyl	X-ray attenuation, bone tissue engineering and imaging	GelMA hydrogel containing 0.16 mM of 60 nm AuNPs exhibited higher contrast during µCT imaging due to X-ray attenuation by AuNPs. Further, GelMA-AuNPs exhibited good cytocompatibility and osteogenic differentiation of mesenchymal stem cells	(Celikkin et al., 2019)
Photodegradable gelatin hydrogels	Azide modification of gelatin	To impart photocleavable property to hydrogel	The azide functionalized gelatin hydrogel enabled the fabrication of micrometer size of hollow structures mimcking blood vessels upon multi-photon excitation process using femtosecond pulsed laser	(Watanabe et al., 2020)
BSA/Gel-SS-PTX/PTX-SS-COOH nanoparticle	Thiolation	Redox sensitive drug (paclitaxel or PTX) delivery system for anticancer therapy	Thiolated gelatin crooslinked with disulfide containing prodrug (PTX-SS-COOH) yeiled redox sensitive amphiphilic polymer which upon self assembly in the presence of BSA formed stable nanoparticles. The nanoparticles exhibited the MMP2- and redox-mediated relase of PTX specially in the tumor microenvironment	(Zhou et al., 2020)
Cross-linked alginate-gelatin hydrogels	Azide modification of gelatin	Cardiac tissue engineering	Bio-orthogonal double cross-linked alginate-gelatin hydrogels supported rapid gelation, biocompatibility and tunable viscoelastic properties and maintained the viability and functional properties of human cardiac fibroblasts (HCFs)	(Testore et al., 2025)
Gelatin-tetrazine-norbornene hydrogel	Amination	Tissue engineering & *in vitro* modeling	Degree of modification (DOM) tuned between 5% and 15% Hydrogels prepared with higher DOM cross-linked faster (10–20 min) compared to hydrogels prepared with lower DOM (60–70 min). Human dental pulp stem cells successfully 3D cultured in the hydrogels (percentage viable cells >85%)	(Contessi Negrini et al., 2021)
Nanosilver/halloysite nanotubes/gelatin methacrylate (nAg/HNTs/GelMA) hybrid hydrogel	Methacryloyl	Bone tissue engineering	Good biocompatibility of nAg/HNTs/GelMA hydrogel against human periodontal ligament stem cells (hPDLSCs) and macrophages Further, nAg/HNTs/GelMA hydrogel modulated inflammatory cytokines secreted by macrophages and enhanced the osteogenic differentiation of hPDLSCs due to the presence of HNTs Moreover, the formulation facilitated broad-spectrum antibacterial ability of implants *in vivo*	(Ou et al., 2020)
Gelatin methacryloyl (GelMA)/beta-tricalcium phosphate (TCP) scaffold	Methacryloyl	Dental tissue engineering	*In vitro* studies indicated good cytocompatibility of GelMA/20% TCP scaffold. GelMA/20% TCP scaffold promoted odontogenic differentiation of dental pulp stem cells (DPSCs). Therapeutic role of the GelMA/20% TCP scaffolds in promoting hard tissue formation was validated in rat model	(Han et al., 2024)
Gelatin methacryloyl (GelMA) hydrogel microspheres	Methacryloyl	Dental tissue engineering	Human dental pulp stem cells (hDPSCs) laden GelMA microspheres prepared by electrostatic microdroplet method with size and Young’s modulus of ∼200 μm and 582.8 ± 66.0 Pa respectively could effectively adhere, spread, proliferate, and secrete extracellular matrix proteins. Moreover, hDPSCs could withstand cryopreservation in the microspheres. *In vivo* (mice) studies corroborated the pulp-like tissue regeneration ability of hDPSCs-laden GelMA microspheres	(Yang et al., 2021)
Gelatin methacryloyl (GelMA) scaffold	Methacryloyl	Oral mucosa tissue engineering	Collagen-based hydrogels were demonstrated to be superior than GelMA hydrogel to support the adhesion/differentiation of epithelial cells and to prmote the viability/growth of fibroblast cells	(Tabatabaei et al., 2020)
Calcium silicate cement/methacrylated gelatin (GelMA) scaffold	Methacryloyl	Dental tissue engineering	3D-printed mineral trioxide aggregates (MTA)-GelMa scaffold was superior than bare GelMa scaffold for promoting the proliferation, adherence, and spreading of human dental pulp stem cells (hDPSCs) and the differentiation of osteocytes	(Choi et al., 2022)
Gelatin-/silver nanoparticle (GT/Ag) composite cryogel	Amination	Wound dressings to promote burn wound repair	GT/Ag cryogel exhibited excellent antibacterial activity against multi-drug-resistant *Staphylococcus aureus* and *Pseudomonas aeruginosa* under *in vitro* conditions GT/Ag cryogel was more effective than Tegaderm™ dressing and conventional GT cryogel for promoting wound contraction, collagen deposition, angiogenesis and reducing inflammation in a *Pseudomonas aeruginosa*-infected burn wound model	(Huang et al., 2022)
Bioink based on methacryl-modified gelatin (GelMA)	Methacryloyl	Bioprining for bone tissue engineering	Bioprinted co-culture constructs comprising methacryl-modified gelatin, hydroxyapatite and adipose-derived stem cells facilitated deposition of bone matrix associated proteins like collagen type I, fibronectin and alkaline phosphatase within the 30-day culture	(Leucht et al., 2020)
Gelatin methacryloyl (GelMA) hydrogel microspheres	Methacryloyl	Tissue engineering	Umbilical cord-mesenchymal stem cells (UC-MSCs) attached to GelMA microspheres promoted the proliferation and migration of fibroblasts UC-MSCs-laden GelMA hydrogel accelerated collagen deposition, angiogenesis, wound healing and skin tissue regeneration in mouse model by regulating wound healing-related factors such as transforming growth factor-β (TGF-β) and type III collagen	(Li et al., 2022)
Fullerene nanocomposites (C60@PDA) loaded methacryloyl (GelMA) hydrogel	Methacryloyl	Tissue engineering	The formulation exhibited free radical scavenging ability, reduced oxidative stress and promoted re-epithelialization, collagen deposition & angiogenesis in rat skin wound model	(Chen et al., 2023)
*Chlorella vulgaris*-loaded separable microneedle (CvMN) with polyvinyl acetate (PVA) substrate and gelatin methacryloyl (GelMA) tips	Methacryloyl	Oxygen-delivering systems for wound healing	Both *in vitro* and *in vivo* results indicated that GelMA laden with CvMN promoted cell proliferation, migration, and angiogenesis and enhance wound healing in diabetic mice effectively through sustained generation of dissolved oxygen	(Zhao et al., 2023)
Graphene oxide (GO)/gelatin nanofibrous scaffolds loaded with N-acetyl cysteine	Thiolation	Antioxidant dressing material for wound healing	The formulation reduced oxidative stress and scar formation and enhanced neovascularization, re-epithelialization and wound healing in mouse excisional wound-splinting model	(Yu et al., 2023)
Dual-crosslinked methacryloyl gelatin (GelMA) and methacryloyl silk fibroin (SilMA) hydrogel	Methacryloyl	Tissue engineering & wound healing	The formulation showed improved biodegradation & mechanical properties, accelerated cutaneous wound healing by facilitating epidermal tissue regeneration & collagen deposition in a full-thickness skin wound model in mice	(Xu et al., 2023)
3D-Bioprinted methacryloyl gelatin (GelMA)/gelatin/amniotic membrane extract (AME) scaffold	Methacryloyl	Tissue engineering	The designed hydrogel loaded with keratinocytes, fibroblasts and human umbilical vein endothelial cells (HUVEC) promoted the formation of well-defined skin layers and the creation of extracellular matrix (ECM)	(Pazhouhnia et al., 2024)
Gelatin-glycidyl methacrylate/graphene oxide (Gel-GMA/GO) composite hydrogels	Methacryloyl	Tissue engineering	The Gel-GMA/GO hydrogel demonstrated excellent biocompatibility, hemocompatibility, and controlled degradability as compared to conventional Gel/GO hydrogel	(Atef et al., 2025)
Methacryloyl gelatin (GelMA) hydrogel	Methacryloyl	Tissue engineering & wound healing	Hydrogel showed excellent biocompatibility, promoted the growth and proliferation of fibroblasts and accelerated wound healing in type I diabetic rats by downregulating pro-inflammatory cytokines	(Liu et al., 2022)
Polypyrrole-modified methacryloyl gelatin (GelMA)-based hydrogel	Methacryloyl	Tissue engineering & wound healing	The prepared GelMA hydrogel laden with neutrophils mimicking nanoparticles showed remarkable efficacy in inhibiting inflammation and preventing tissue adhesion by modulating the expression of both inflammatory and tissue adhesion-related factors in a mouse model of intestinal perforation	(Han et al., 2025)
Nanocomposite hydrogel of thiolated gelatin/gelatin methacrylate/Laponite®	Thiolation & Methacryloyl	Tissue adhesive	The designed hydrogel exhibited hemostatic and self-adhesive properties and promoted fibroblast (L929 cells) proliferation. Further, the designed hydrogel showed significantly less blood clotting time than the hydrogel without laponite	(Rajabi et al., 2020)
Methacrylated gelatin matrix (GelMA) crosslinked with phenylboronic acid and imidazole ions	Methacryloyl	Wound healing	GelMA hydrogel crooslinked with with phenylboronic acid and imidazole ions displayed rapid gelation (3 s), low swelling properties (1.07 g/g), robust tensile strength (56.83 kPa) and high adhesive strength (63.38 kPa). The hydrogel demonstrated effective healing of oral ulcers in the diabetic rat model by redcing oxidative stress and inflammation in the ulcers	(Chen et al., 2025)
Thiolated gelatin hydrogel	Thiolation	Tissue engineering	Hydrogel injection into mouse model of intracerebral hemorrhage (ICH) reduced neuron loss, attenuated the neurological deficit post-operation, decreased the activation of the microglia/macrophages and astrocytes and promoted functional recovery of ICH	(Xu et al., 2020)
Thiolated gelatin microparticles (GMPs)	Thiolation	Contrlled drug delivery & tissue enginerring	Thiolated GMPs demonstrated better biocompatibility and robust platform for mesenchymal stem cell attachment than non-thiolated GMPs Thiolated GMPs preserved the cytocompatibility of gelatin while enabling post-fabrication modification with other utility group for tissue engineering application	(Pearce et al., 2021)
Selenium functionalized gelatin (GelSe) hydrogel	Selenation	Regenerative medicine & tissue engineering	GelSe hydrogels facilitated the proliferation of normal human dermal fibroblasts (NHDFs) and maintined the optimal viability of human pluripotent stem cell-derived cardiomyocytes (hiPSC-CMs) compared to gelatin methacryloyl (GelMA). GelSe also exhibited antioxidant and antimicrobial capacities, particularly against *E. coli* and *S. aureus*	(Pérez-Araluce et al., 2024)
Poly(N-isopropylacrylamide) (PNIPAAm)-grafted gelatin	Amination	Ophthalmic drug delivery carriers	The amination degree of gelatin dictated the structure-property-function relationships for injectable depot formulation of pilocarpine Increase in amination degree facilitated extended drug release profiles and provided significant therapeutic benefits	(Morimoto et al., 2008)
Hybrid gelatin methacryloyl (GelMA)/phenyl isothiocyanate-modified gelatin (Gel-Phe) bioadhesive	Methacryloyl	Dental tissue engineering	GelMA/Gel-Phe (1:1–1:15 w/w) bioadhesive showed low cytotoxicity, a highly porous network, and remarkable hemostatic properties towards fast blood absorption and rapid clotting effect *In vitro* porcine skin bleeding and an *in vitro* dental bleeding model confirmed the ability of bioadhesive to reduce the blood clotting time by 45%	(Chang et al., 2021)
Pegylated gelatin nanoparticles	PEGylation	Controlled drug delivery	The PEGylated paclitaxel (PTX)-loaded nanoparticles showed the desired PTX release kinetics, inhibited the proliferation of non-small cell lung cancer cell lines (L132 and 293T) and showed the highest pulmonary deposition and antitumor activity in the tumor bearing mice model.	(Gu et al., 2021)
Diselenide functionalized gelatin (G-Se-Se-G) hydrogel	Selenation	Wound healing	G-Se-Se-G gel reduced nitric oxide (NO) donor such as S-nitroso-N-acetylpenicillamine (SNAP) by glutathione peroxidase-like mechanism to produce NO Murine studies confirmed that topically applied G-Se-Se-G gel and SNAP promoted faster epithelization and collagen deposition at the wound site	(Das et al., 2023)
Redox responsive diselenide gelatin (G-Se-Se-G) nanoparticles	Selenation	Controlled drug delivery	Doxorubicin loaded seleno-gelatin (G-Se-Se-G) nanoparticles showed selective toxicity towards lung cancer (A549) cells Intracellular drug release studies confimed the redox mediated preferential release of Dox from G-Se-Se-G carrier in A549 cells *In vivo* studies using A549 derived xenograft tumor model established the safety and efficacy of Dox loaded G-Se-Se-G nanocarrier	(Das et al., 2024)

**FIGURE 5:**
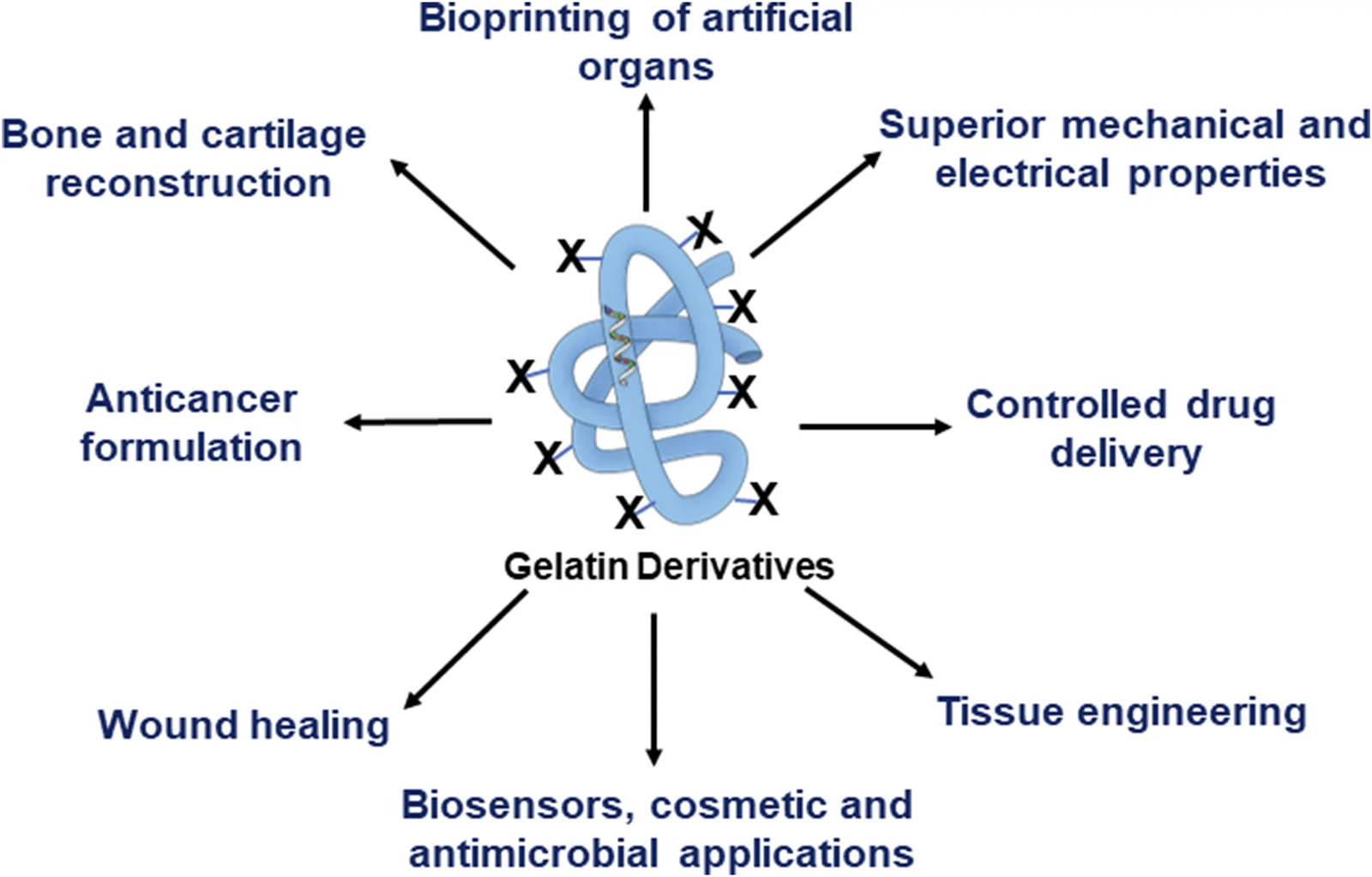
Schematic shows the usage of chemically modified gelatin in various biomedical applications.

These studies, together justify the growing utility of gelatin derivatives for a variety of biomedical applications such as controlled drug delivery, tissue engineering, wound healing, anticancer treatment, blood clotting, immunomodulation, bone and cartilage reconstruction, fabrication of biosensors, bioprinting of artificial organs, cosmetic and antimicrobial applications Indeed, biomaterials fabricated using gelatin derivatives have been demonstrated to maintain higher cellular and tissue viability (including dental pulp stem cells, mesenchymal stem cells, hepatocytes, fibroblasts, cardiomyocytes, and osteoblasts) while also promoting cell proliferation and differentiation. Overall, chemical derivatization of gelatin with varying degree of substitution offers a novel oppurinity to tune its mechanical, electrical, rheological and tactile properties, thereby facilitating its interaction with cellular systems without compromising its biocompatibility.

## 6 Future recommendation

The inherent heterogeneity of gelatin in terms of molecular weight and surface charge raises serious concerns regarding the consistency of batch-to-batch product quality (Milano et al., 2023; Zhang and Wang, 2024; Mikhailov, 2023; Montazerian et al., 2023). This limitation can be overcome by using recombinant gelatin as a starting material for chemical modifications (Olsen et al., 2003; Gellermann et al., 2019; Duan et al., 2011; Song et al., 2024). The recombinant DNA technology ensures the production of gelatin of a fixed molecular weight containing the desired amino acid sequence from a desired source (Figure 6). Additionally, this technology also circumvents other major disadvantages of traditional methods, such as the risk of pathogenic transmission and unwanted extraction-, as well as processing-induced changes in the backbone of gelatin. The basic principle of this technology involves cloning a segment of gene encoding collagen from a desired species into expression vector under the control of an inducible promotor followed by expression and purification of the protein using a suitable host system (Figure 6). The initial studies in this regard involved optimising the methodology for producing gelatin using a yeast expression system wherein the genetic sequence encoding a segment of rat collagen was cloned into *Pichiapastoris* under the control of methanol-inducible alcohol oxidase (AOX1) (Gellermann et al., 2019). One of the limitations of using *Pichiapastoris* as the expression system was the production of gelatin with non-hydroxylated proline due to the lack of prolyl 4-hydroxylase (P4H), an enzyme responsible for post-translational modification of proline residues. Subsequently, significant progress has been made in producing native gelatin with hydroxylated proline by co-expressing the genetic sequence of gelatin and of P4H under two different inducible promoters in the same host *Pichiapastoris* (Duan et al., 2011). This strategy has ensured the production of gelatin in the native form with hydroxyproline restudies at desired positions. Further, recombinant DNA technology can also be used to produce gelatin of human origin (Song et al., 2024). Human-derived gelatin gains significance as it ensures the immunocompatibility of delivery systems for human use. Notably, recombinant gelatin has already been introduced in to market, but its cost-effectiveness is still a major issue. With quantum advancements in the field of molecular biology in recent years, it is expected that the methodology of producing recombinant gelatin will become economically viable in the near future. Another important limitation related to the chemically modified derivatives of gelatin is that there is no information on the effect of various chemical modifications on the structural organization of gelatin. As chemical modification leads to alteration in the availability of various functional groups, it is expected to influence the structural organisation of gelatin by affecting the status of inter and intrachain hydrogen bonding. Indeed, as discussed previously, aminated gelatin is less efficient than native gelatin to form a gel like network as the presence of amine group hinders the formation of inter and intrachain hydrogen bonding (Hayashi et al., 1990). Accordingly, future studies are needed to understand the effect of other types of chemical modifications such as thiolation, PEGylation, selenation and azidation among others on the formation of various secondary structures of gelatin (Figure 6). Further, neither gelatin nor of its any chemical derivatives *per se* have been explored as therapeutic agent until now. Since some of the functional groups used for gelatin derivatization, such as thiol and selenium, are well reported for various pharmacological activities (Jain and Priyadarsini, 2017; Gallo-Rodriguez and Rodriguez, 2024; Chuai et al., 2021), it is anticipitated that gelain functionalized with thiol and/or selenium may *per se* act like a therapeutic agent. Accordingly, it would be interesting in future to examine the therapeutic potential of thiolated and selenated gelatin against various disease models by employing cellular and *in vivo* model systems (Figure 6). Furthermore, it is also worth investigating whether gelatin, following chemical modification, elicits any immune response. In order to address this, the chemically modified derivatives of gelatin should be thoroughly examined for acute and chronic toxicities using *in vivo* models. Lastly, an attempt should be made to create unique structural forms (tissue and organ printing) using gelatin and its derivatives to fully utilize their potential for biomedical applications (Figure 6).

**FIGURE 6:**
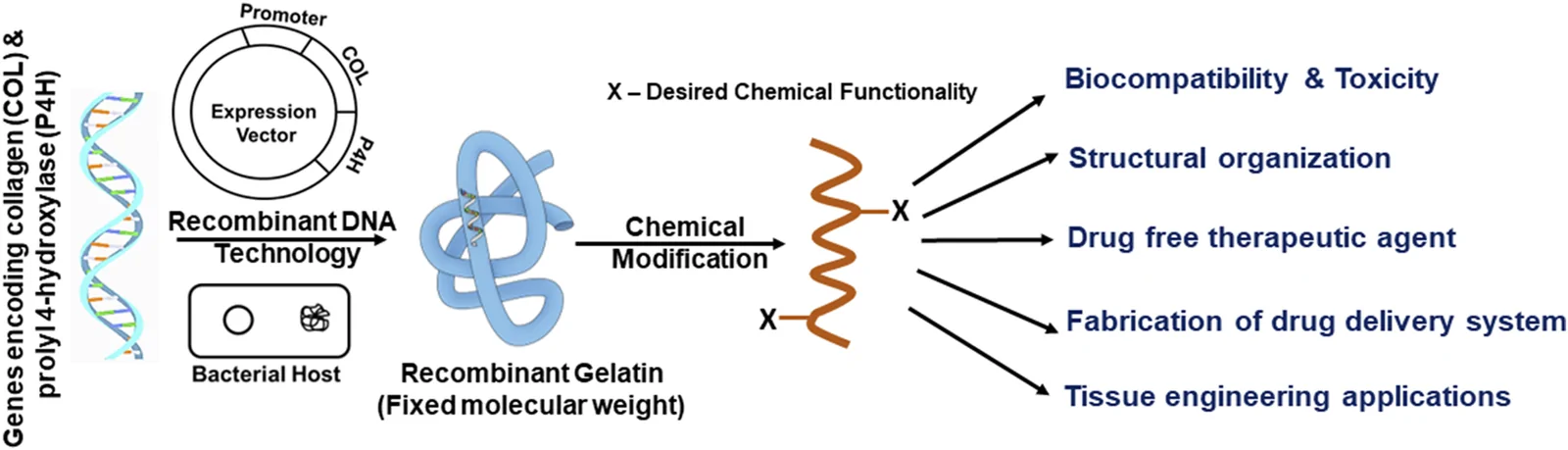
Scheme depicts the proposed future strategies to fully utlize the potential of chemically modified derivatives of gelatin.

## 7 Conclusion

The scope of chemical modification of gelatin is expanding rapidly. The present manuscript has reviewed particularly on the amine, thiol, acrylate, PEG, azido, and selenium derivatives of gelatin. However, considerable research has also been conducted on the modification of gelatin with several other chemical moieties including but not limitated to carbohydrates and polyphenols to impart bioactivity. A detailed discussion of these gelatin derivatives is beyond the scope of the present review article and is available elsewhere. Nevertheless, studies to date have established that chemical modification of gelatin can significantly improve its physicochemical properties and biodegradability making it suitable for various biomedical applications.
